# All WSe_2_ 1T1R resistive RAM cell for future monolithic 3D embedded memory integration

**DOI:** 10.1038/s41467-019-13176-4

**Published:** 2019-11-15

**Authors:** Maheswari Sivan, Yida Li, Hasita Veluri, Yunshan Zhao, Baoshan Tang, Xinghua Wang, Evgeny Zamburg, Jin Feng Leong, Jessie Xuhua Niu, Umesh Chand, Aaron Voon-Yew Thean

**Affiliations:** Department of Electrical and Computer Engineering, National University of Singapore, 4 Engineering Drive 3, Singapore, 117583 Singapore

**Keywords:** Electrical and electronic engineering, Electronic devices, Electronic and spintronic devices

## Abstract

3D monolithic integration of logic and memory has been the most sought after solution to surpass the Von Neumann bottleneck, for which a low-temperature processed material system becomes inevitable. Two-dimensional materials, with their excellent electrical properties and low thermal budget are potential candidates. Here, we demonstrate a low-temperature hybrid co-integration of one-transistor-one-resistor memory cell, comprising a surface functionalized 2D WSe_2_ *p-*FET, with a solution-processed WSe_2_ Resistive Random Access Memory. The employed plasma oxidation technique results in a low Schottky barrier height of 25 meV with a mobility of 230 cm^2^ V^−1^ s^−1^, leading to a 100x performance enhanced WSe_2_ *p*-FET, while the defective WSe_2_ Resistive Random Access Memory exhibits a switching energy of 2.6 pJ per bit. Furthermore, guided by our device-circuit modelling, we propose vertically stacked channel FETs for high-density sub-0.01 μm^2^ memory cells, offering a new beyond-Si solution to enable 3-D embedded memories for future computing systems.

## Abstract (web summary)

Designing efficient, scalable and low-thermal-budget 2D Materials for 3D integration remains a challenge. Here, the authors report the development of a hybrid-(solution-processed-exfoliated) integration of 2D Material based 1T1R which uses a multilayer WSe_2_ *p*-FET and a multilayer printed WSe_2_ RRAM.

## Introduction

Emerging non-von Neumann architectures with intensive in-memory computing like next-generation deep learning and neuromorphic chips will demand high-density integration of embedded memory. Three-dimensional monolithic (sequential) multilayer stacking of transistors and memory among interconnects may allow us to expand the on-chip memory density. Such architectures will not only overcome the two-dimensional (2D) die limitations but also enable new three-dimensional (3D) computation systems, where logic and memory elements are intimately co-located, to significantly improve the memory access bandwidth and energy^1^. However, to fully realize such 3D systems, there are fundamental technology challenges to overcome. Among which, transistor-interconnect thermal budget incompatibility poses a major road block. Advanced low-resistivity copper interconnect with low-*k* dielectric interlayer cannot tolerate thermal exposure above 400 °C^2^. Since the thermal activation of dopants in Si-based devices are typically between 600 and 1000 °C, Si transistor formation below such temperature results in device performance and reliability degradations^3^. This low thermal budget technology barrier calls for both material and process integration breakthroughs, to enable new platforms for 3D integration.

Carbon nanotubes (CNTs) field-effect transistor (FETs) and 2D semiconducting van der Waal-layered crystals (2DMat) have drawn immense attention as transistor channel material, due to their intrinsic performance that rivals silicon, as well as upcoming successors like germanium, silicon germanium, and III–V compound semiconductors at sub-nanometer channel thickness regime^4^. More importantly, the potential of such nanomaterials for low-temperature, large-area transfer and integration, independent of their material synthesis^5,6^, puts them in an advantageous position to be co-integrated additively with metal interconnects on CMOS (complementary metal–oxide–semiconductor) chips (Supplementary Table 1). The feasibility of 3D integration with CNTs has already been demonstrated by Shulaker et al.^1^, but that of 2DMat has only started to gain traction^7^. 2DMat, with their intrinsic nanolayer structures and variety of electronic structures are expected to add more functionalities for process temperature-limited technologies like sequential/monolithic 3D chips^8^ and high-performance flexible electronics^9^.

In this work, we demonstrate the feasibility of hybrid co-integration of a surface-engineered WSe_2_-based thin film transistor (TFT) and resistive random access memories (ReRAM) to realize a 1 transistor–1 resistor (1T1R) memory cell. This is done through integrating WSe_2_ of different morphologies (single crystalline for TFT, and polycrystalline for ReRAM) processed through different synthesis technique, to address the conflicting charge transport attributes required for logic and memory. As TFT should be optimized for high performance and low leakage, the high-quality mechanically exfoliated WSe_2_ is utilized as the transistor channel. On the other hand, ReRAM should be optimized for low-voltage defect-enabled switch ability, for which solution-processed WSe_2_ is employed. Despite WSe_2_ 2DMat being well investigated for future logic application, its application for 1T1R memory cell by hybrid co-integration is yet to be investigated. Moreover, our proposed processes are room temperature based, offering compelling compatibility with temperature-limited 3D monolithic process integration and flexible electronics processing. Furthermore, we propose through calibrated compact device modeling and circuit simulations that sub-0.01 µm^2^ 1T1R cells with good read/write margins are feasible by stacking 2D nanosheets to realize a multiple-stacked 2D TFTs to drive the 2D ReRAMs.

## Results

### WSe_2_ select transistor material

With a large bandgap, a reasonably high intrinsic thin-channel carrier mobility^10^, and *n*–*p* polarity that can be easily modulated by contact Schottky barrier metal^11^, WSe_2_ offers great potential for low leakage and performant CMOS logic gates^12^. The low on-state resistance and off-state leakage potential of the WSe_2_ transistor also makes them a good select transistor candidate for 1T1R memories, which calls for minimization of voltage loss across the transistor during memory cell set/reset and the off-state sneak current in the array, respectively. Despite the favorable intrinsic attributes, WSe_2_ transistors are still challenged by extrinsic degradations in mobility and high contact resistance. The reports of WSe_2_ exhibiting high mobility at low temperatures^13^ suggest the detrimental role played by various scattering sources, such as phonons, Coulomb impurities (CI), and intrinsic defects in mobility degradation. Although passivation methods based on dielectric deposition, including atomic layer-deposited high-*k* encapsulation^14^, have been pursued, the process uniformity remains challenging due to undesired grain boundary nucleation^15^. Thus, it becomes necessary to investigate other strategies including uniform native oxide passivation solution as well.

In addition, minimizing transistor access resistance is essential to translate the performance gains from channel carrier mobility. While heavy source/drain (S/D) doping is the most preferred method for improving contact resistance in conventional Si devices, such substitutional doping in 2DMat comes at the expense of increased defect density^16^. For 2DMat, several approaches ranging from material modification to the co-integration of graphene electrodes^17^ have been proposed. However, they present new challenges in stability and work-function limitations. For example, the semiconducting 2H phase to metallic 1T phase modification^18^ can improve contact resistance significantly, but the low-temperature stability and Fermi level to conduction band alignment limits its utilization for *p*-FETs^14^. Graphene contacts, due to its Fermi level alignment close to the conduction band, would also lead to undesirable electron injection for *p*-FETs^17^. In this work, we concurrently address strategies for hole carrier doping, mobility enhancement, Schottky barrier, and contact resistance reduction through a single-step process that overcomes the issues of stability and *p*-contact work-function alignment. We developed a self-limiting single-step, low-temperature WO_3_ formation on channel surface and under the S/D contacts by post-contact remote plasma oxidation. This process simultaneously enhances the WSe_2_ TFT mobility by almost 76 times and reduces the contact resistance by a hundred-fold. By implementing Ag-WO_3_-WSe_2_ metal–insulator–semiconductor (MIS) contact, we achieved an ultra-low Schottky barrier height (SBH) of 25 meV with respect to the WSe_2_ valence band, significantly enhancing hole injection.

### Low-temperature surface layer plasma oxidation for WSe_2_ FET

**Fig. 1:**
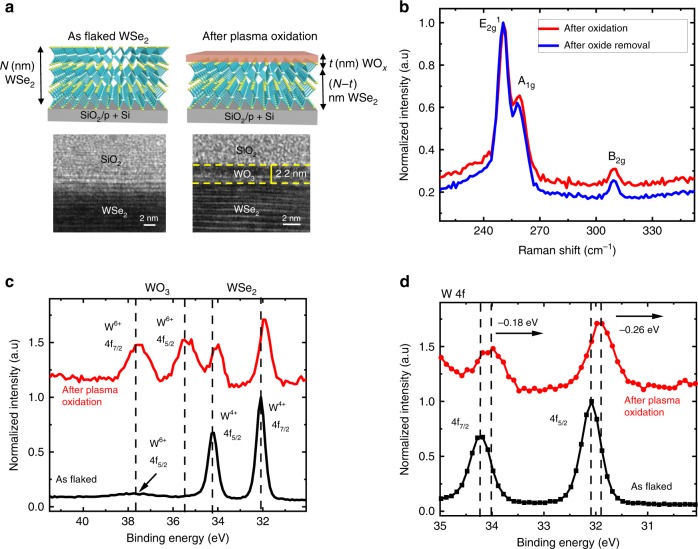
Remote plasma oxidation process and characterization. **a** Schematic representation of surface plasma oxidation and the corresponding cross-sectional transmission electron microscope (xTEM) images. The xTEM image of oxidized WSe_2_ shows 2.2 nm of WO_3_ upon oxidation, which is a consumption of three layers of WSe_2_. **b** Raman spectroscopy comparison before oxidation and after oxide removal, in order to have comparison between WSe_2_ of same thickness. No apparent change in peak position is observed, implying no crystalline damage due to plasma oxidation. **c** X-ray photoelectron spectroscopy (XPS) comparison of as flaked WSe_2_ and plasma-oxidized WSe_2_. The appearance of two additional peaks after oxidation corresponds to an *x* factor of 3 in WO_*x*_. **d** W 4f core level XPS spectrum comparison of pristine WSe_2_ and plasma-oxidized WSe_2_. The observed shift to lower binding energy implies electron transfer from WSe_2_ to WO_3_

For 2D transition metal dichalcogenides (TMDs), the thickness is a critical parameter influencing their electronic and optical properties. Although mechanical exfoliation results in high-quality WSe_2_ flakes, the approach does not allow for precise thickness control. Considerable amount of research has been devoted to realizing a thickness reduction strategy, such as the use of focused ion beam^19^, ozone treatment^20,21^, XeF_2_ vapors^22^, plasma oxidation^23^, thermal oxidation^24^, and so on. However, these approaches can induce minor^22^ as well as major damage to the crystallinity of the WSe_2_ material with resultant negative impact to its electrical performance. While the above-mentioned reports focus on oxidation as a thickness reduction strategy for mechanically exfoliated samples, we introduce a low-temperature remote plasma oxidation process (Methods section) and study the utility of the formed oxide as a MIS contact and encapsulation layer using detailed material and electrical characterization. We show that gentle plasma oxidation can create a layer of surface WO_*x*_, which do not damage the underlying WSe_2_ structure. Figure 1a shows the cross-sectional transmission electron microscope (xTEM) image of the WSe_2_ flake before and after the remote plasma oxidation, from which the presence of WO_*x*_ and the quality of exposed WSe_2_ are confirmed. The thickness of the formed WO_*x*_ is ~2.2 nm for a consumption of three layers of WSe_2_, as confirmed by xTEM. Irrespective of oxidation time, the WO_*x*_ formation is found to be self-limiting as well (Supplementary Fig. 1). The same oxide thickness has been validated for WSe_2_ of different starting area and thicknesses under the same oxidation condition.

Raman spectroscopy and X-ray photoelectron spectroscopy (XPS) analysis were done to determine the nature of WO_*x*_ formed by this process. As the vibrational and optical properties strongly vary with thickness, a comparison of “oxidized WSe_2_” and “oxide-removed WSe_2_” has been performed. The oxide removal process, which is selective to WSe_2_, is done using KOH solution (Methods section). From the Raman spectrum in Fig. 1b, we observe the typical out-of-plane A_1g_ mode, in plane E_2g_ mode and the bulk, B_2g_ mode for two prepared four-layer WSe_2_ samples; one with WO_*x*_ (after oxidation) and one without WO_*x*_ (after oxide removal). No apparent Raman peak shift is detected between the two samples—ruling out the presence of any plasma oxidation induced stress in WSe_2_. The resultant WO_*x*_ appears to be amorphous due to the absence of the 800 cm^−1^ signature peak, indicating crystalline WO_*x*_^25^ (Supplementary Fig. 2). The amorphous WO_*x*_ structure is further corroborated by the xTEM images (Fig. 1a), which did not reveal any crystalline order in the WO_*x*_ layer. Since there exists reports of crystalline WO_3_ formed through air heating at higher temperature of 400 °C^25^, the amorphous WO_*x*_ is likely due to our low-temperature plasma oxidation process. From the XPS analysis in Fig. 1c, we confirm the stoichiometry of amorphous WO_*x*_ to be native WO_3_. Specifically, peaks at 35.5 and 37.7 eV after plasma oxidation correspond to the binding energies of W^6+^, indicating the presence of WO_3_^25^. Furthermore, we observe charge transfer mediated by WO_3_ from the XPS spectrum after plasma oxidation. The observed decrease in binding energy of W 4f core levels (0.18 eV reduction in W^4+^ 4f_7/2_ and 0.26 eV reduction in W^4+^ 4f_5/2_) (Fig. 1d) suggests that there is electron transfer from WSe_2_ to WO_3_. This is attributed to the high work function of WO_3_ consistent with other reported studies^22^_._ We show here that low-temperature plasma oxidation, capable of self-limiting to ~2.2 nm amorphous WO_3_, produces an ultra-thin hole donor layer that is also gentle to WSe_2_.

**Fig. 2:**
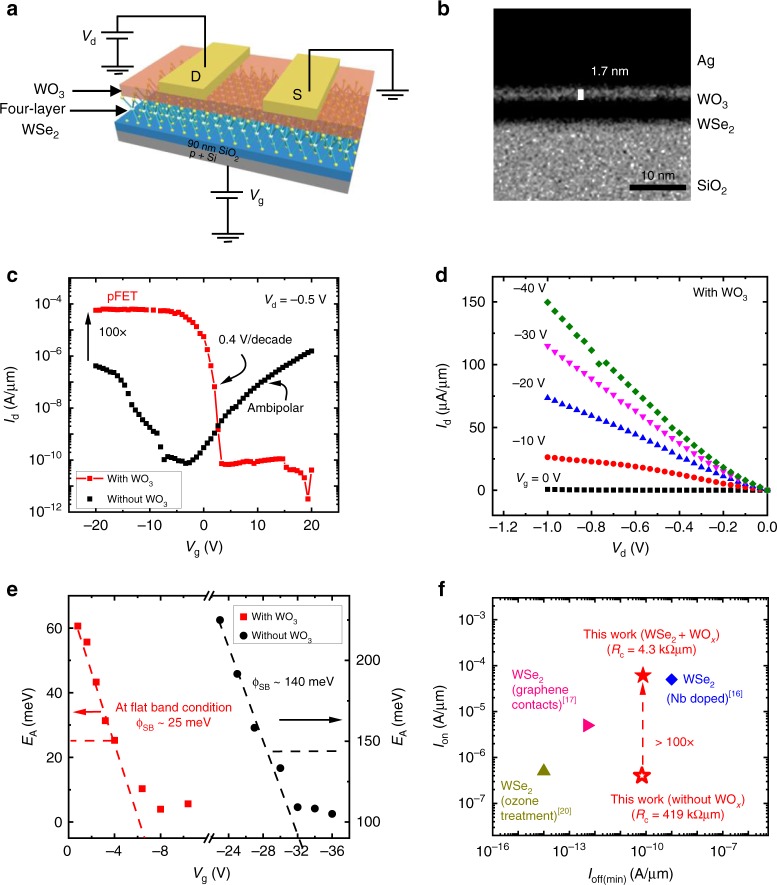
Surface plasma-oxidized WSe_2_ TFT and electrical characterization. **a** Device schematic showing a four-layer WSe_2_ and 2.2 nm WO_3_ on SiO_2_/*p* + Si layer with gate length (*L*_g_) = 1.80 µm and width (*W*) = 2.05 µm. **b** Transmission electron microscopy image of the device contact region, after post-contact plasma oxidation, revealing the presence of WO_3_ underneath the metal contacts**. c** *I*_d_*–V*_g_ plots for four-layer thick device with and without WO_3_. **d** *I*_d_*–V*_d_ characteristics after plasma oxidation for different gate voltages. **e** Effective Schottky barrier height extraction from low-temperature transfer characteristics and Arrhenius plot. At flat band condition, the curve deviates from linearity and the corresponding activation energy becomes the Schottky barrier. **f** Benchmark plot showing the performance of plasma-oxidized *p*-FET versus other reported data. *I*_on_ is determined at *V*_d_ = −0.5 V

Figure 2a shows the schematic of the fabricated device consisting of a four-layer WSe_2_ and three-layer WO_3_. The detailed fabrication procedure can be found in the Methods section. In order to realize a thinner WO_3_ layer under the Ag S/D contacts to minimize tunneling resistance, we chose to perform post-contact plasma oxidation. The key advantage of this strategy is that the WO_3_ growth rate under the S/D region would be moderated by the metal contact. The TEM image (Fig. 2b) confirms thinner (1.7 nm) WO_3_ layer under the contact as opposed to the thicker (2.2 nm) oxide formation for the exposed channel, despite the common plasma oxidation process. Due to limited diffusion of O radicals at the Ag-WSe_2_ terminations on both ends of the electrodes, the O radicals can only propagate laterally under the Ag contacts, resulting in a reduced thinning of WSe_2_ layer^24^ under the contact metal as opposed to the exposed channel regions.

We characterized the resultant TFT performance by measuring the transfer and output characteristics (Fig. 2c, d). Figure 2c compares the transfer characteristics with and without post-contact plasma oxidation. The devices without WO_3_ is unremarkable, showing ambipolar conduction, with slightly stronger n-type (*V*_g_ >−5 V) than *p*-type conduction (*V*_g_ <−5 V). Upon plasma oxidation, the device exhibits strong *p-*type conduction. Most remarkably, a 100× enhancement in the hole current is accompanied by a strong polarity change, where n-type conduction is completely suppressed. To further investigate the TFT performance improvement, we carefully characterized the influence of WO_3_ on mobility and contact resistance.

We measured the gate inversion capacitance of the oxidized device and found it to have increased by 2× compared to the geometrical value (77 vs. 38 nF cm^−2^). Details of inversion capacitance extraction are found in Supplementary Fig. 3 and Supplementary Note 1. Since we do not observe CV frequency dispersion (Supplementary Figure 3e) that indicates significant fast or slow charge trapping/de-trapping processes, we exclude the possibility of spurious charges and disorder at WSe_2_ bottom-gate dielectric interface as reported by Pradhan et al. ^13^. Instead, we believe the interfacial charge transfer at the WSe_2_-WO_3_ heterostructure contributed towards the capacitance increase. To ensure accurate mobility extraction, we emphasize here the need for CV measurements, instead of making capacitance assumptions based on geometry. From Fig. 2c and the measured inversion capacitance, we extracted a dramatic 76× hole field-effect mobility (*µ*_FE_) increase from 3 cm^2^ V^−1^ s^−1^ (non-oxidized) to 230 cm^2^ V^−1^ s^−1^ and observed a significant 100× reduction of contact resistance (*R*_c_) to 4.3 kΩµm from our control with 420 kΩµm, which is extracted using the well-reported *R*_total_*–V*_g_ method^26^. The details of field-effect mobility and contact resistance extraction can be found in the Supplementary information (Supplementary Figs. 4 and 5). The *R*_c_ reduction correlates to a considerable lowering of the contact’s SBH to 25 meV with respect to the SBH of 140 meV of our control sample without WO_3_ as shown in Fig. 2e. It appears that the thin WO_3_ under the Ag contact unpinned the contact Fermi level with respect to WSe_2_, closer to the valence band minimum of WSe_2_, owing to the high work function of WO_3_^27^. This would also explain the observed suppression of electron current, as the SBH for electrons would be large. Our room temperature, low-power remote plasma oxidation treatment allows a gentler process to achieve less damage to the underlying WSe_2_ flake, as evident by the non-reduction of the PL signal^23^ (Supplementary Fig. 6) as compared to other reported methods^28^. In addition, the plasma process allows the formation of a uniform thin layer of WO_3_ beneath the contact, which has not been reported. The argument is supported by the observed SBH to be 10× lower than the barrier height reported from other work involving similar surface functionalization with WO_3_^20^_._

Furthermore, we conducted an experiment, where the plasma oxidation was performed prior to contact formation, leading to a uniform thicker (2.2 nm) WO_3_ under S/D contacts and over the channel (Supplementary Fig. 7). While the drive current slightly improved compared to non-oxidized device, the performance is weaker than the post-contact-oxidized sample due to higher contact resistance, which is comparable to the device without oxidation (Supplementary Fig. 5). This suggests the importance of controlling WO_3_ thickness, as a tunneling layer—the thicker WO_3_ with pre-contact oxidation actually degrades the contact resistance due to increased tunneling resistance^29^. Figure 2f benchmarks selected top-performing WSe_2_ devices from various reports. Our work shows the strongest *I*_on_ performance for devices with sub-nA µm^−1^-level *I*_off_min_, showing an extraordinary 100× drive current enhancement with respect to our non-WO_3_ control (Supplementary Table 2).

### WSe_2_ ReRAM material, fabrication, and characterization

2DMat-based ReRAM on multilayer hBN^30,31^, solution-processed multilayer 2D ReRAM^32–35^, MoS_2_ phase change memristor behavior^36^, novel resistive switching approaches such as gate tunable non-volatile resistive switching in monolayer MoS_2_ via atomic re-arrangement of grain boundaries^37^, and fast switching operation enabled by electric field-induced structural transition in MoTe_2_ and Mo_1 − *x*_W_*x*_Te_2_^38^ have been demonstrated. Here, we investigate the potential of a Ag-WSe_2_-Ag ReRAM comprising of a solution-processed WSe_2_ as the resistive memory element and Ag as electrodes, realized using a high-precision Aerosol Jet printing (Methods section and Supplementary Table 3). Apart from being compatible with 3D monolithic integration, the solution-processed approach combined with the aerosol jet printing is chosen to leverage on the in situ sonication-induced modulation of defects in the switching layer through ink quality to study the device impact due to different WSe_2_ morphologies^39^. Compared to traditional metal oxide-based ReRAMs, the realization of forming-free operation with lower switching voltage and current is one of the defining advantages of solution-processed WSe_2_ ReRAM. This may be due to the defect formation and migration with respect to the flake morphology as opposed to shorted metallic conductive bridges in oxide ReRAM^40^. Together with the unique material properties, we demonstrate Ag/WSe_2_/Ag ReRAM that exhibits non-volatile, forming-free, sub-1 V switching characteristics at a set current ≤5 µA, with a low switching energy of 2.9 pJ per bit.

**Fig. 3:**
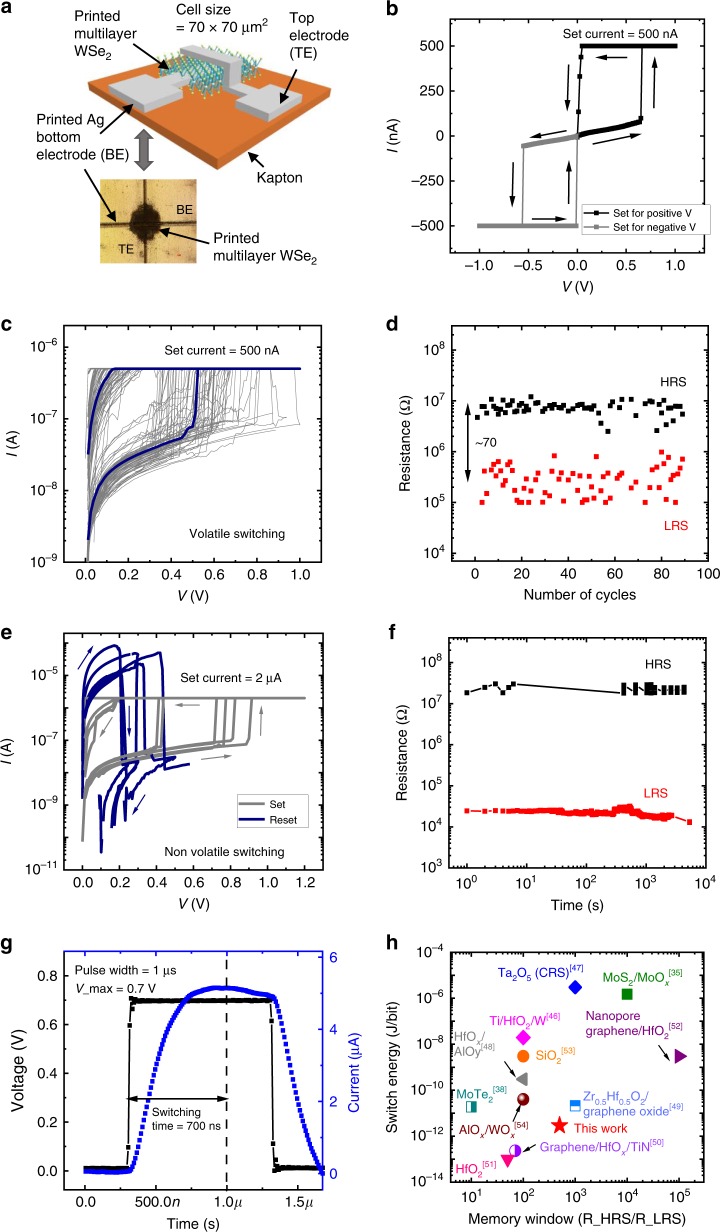
Printed WSe_2_ ReRAM electrical characterization. **a** Schematic of printed WSe_2_ ReRAM with Ag contacts along with microscope image of printed ReRAM. **b** The device sets from HRS to LRS for both positive and negative voltages. **c** Stress cycling data for 90 cycles at a smaller set current of 500 nA, where volatile behavior is observed. **d** Endurance properties of ReRAM at read voltage of 50 mV and a set current of 500 nA. **e** Set and reset operation with a larger set current of 2 µA, exhibiting non-volatile behavior. **f** Retention plot showing LRS and HRS stability till 10^4^ s at a read voltage of 50 mV and set current of 5 µA. **g** Switching time characterization with an AC pulse of 0.7 V amplitude and 1 µs pulse width. **h** Benchmark plot of switching energy per bit vs. memory window of printed WSe_2_ ReRAM with other representative non-volatile resistive switching publications

Figure 3a shows the schematic and the optical microscope image of the WSe_2_ ReRAM with Ag contact. We performed a detailed material characterization using scanning electron microscopy (SEM), Raman spectroscopy and X-ray diffraction (Supplementary Fig. 8). As observed from SEM images, the morphology of the as-printed WSe_2_ layer is highly disordered with randomly distributed clusters, significantly different from the exfoliated-transferred WSe_2_ for the TFT. Raman analysis shows that the E_2g_ mode of the printed WSe_2_ is consistent with the exfoliated WSe_2_. The absence of interlayer coupling-B_2g_ mode and the out-of-plane A_1g_ mode is likely due to the disordered morphology of the printed WSe_2_. The non-orientated switching layer morphology is desired for the vertical memory element as we seek to promote volume-based vacancy or filamentary switching for our devices. Figure 3b shows the direct current (DC) sweep characteristics over a voltage range of −1 to 1 V with a set current limit to 500 nA. The device exhibits forming-free behavior, which can be set in both positive and negative polarity bias. We observe an abrupt switching at sub-1 V set voltage, indicating filamentary-based conduction. Under a set current of 500 nA, the switching characteristic is found to be volatile, that is, the low resistance state (LRS) decays quickly to high resistance state (HRS) after the bias is removed. Figure 3c shows the DC stress cycling of the device over 90 cycles, while Fig. 3d shows the repeatability of the HRS/LRS over the 90 cycles at a read voltage of 50 mV. The device achieves an average HRS/LRS window of ~70 over all the cycles tested. When the set current is increased to 2 µA, the device transitions to a non-volatile switching state. We believe that with the larger set current, the filament thickens and remains stable without external bias^41^. The ReRAM exhibits a unipolar switching behavior where set and reset voltages share the same polarity. As observed in many of the unipolar ReRAM, the reset operation is dominated by the thermophoresis effect^42^, where joule heating ruptures the filament, thus returning the device to the HRS state. The reset voltage ranges from 0.2 to 0.3 V, while the reset current lies in between 80 and 100 μA, as shown in Fig. 3e. The observation of the unipolar switching characteristics is concomitant with that of chemical vapor deposition-grown WSe_2_ reported by Ge et al^43^. We observe a larger memory window of 10^3^ when the set current is increased to 5 µA and achieves a retention time of >10^4^ s (Fig. 3f).

We have confirmed that the switching is not due to Ag ion diffusion in WSe_2_ by comparing the ReRAM switching behavior of otherwise identical devices with inert carbon-based electrodes (Supplementary Fig. 9 and Supplementary Discussion 1), which show similar abrupt switching characteristics. This indicates that the switching mechanism is intrinsic to the WSe_2_ switching layer, likely due to selenium vacancies, thus ruling out the possibility of Ag metal ion conductive-bridge-based mechanism. The switching time, as calculated by applying a voltage pulse of amplitude 0.7 V and 1 μs width is found to be 700 ns (Fig. 3g). With the trade-off existing between programing voltage and switching time^44^, we have chosen to limit the programming voltage to achieve low set power, which results in slower switching time of 700 ns. From the material/structural point of view, controlling the flake size and thickness of the switching layer would be areas that could potentially offer improvement in switching speed^45^. Our devices show one of the lowest reported switching energy (Supplementary Fig. 10, Supplementary Table 4, and Supplementary Note 2) relative to other 2DMat^35,38^ and other oxide-based ReRAMs^46–54^, as illustrated in the Fig. 3h with endurance comparable to other reported 2DMat ReRAMs (Supplementary Table 5). We suspect that the low switching energy is promoted by the excess defects and grain boundaries in our printed WSe_2_ layer. We performed repeatability check for ReRAM devices fabricated across several batches at different times, where we observed consistent switching characteristics, as shown in the cumulative probability distribution plot for set voltage, reset voltage, and ReRAM resistance (Supplementary Fig. 11). We believe that there is still significant opportunity for improvement with respect to ReRAM endurance and other metrics by engineering the flake sizes with the solution-processed approach.

Although Aerosol jet printing technique allows additive deposition of inks with a wide range of viscosities to realize quick prototyping of devices at relaxed dimensions (down to 10 μm feature size), it is not a suitable method for industrial-scale production because of the low throughput and large feature size achievable. Except for the low thermal budget of our process, we do not believe that our additive approach would significantly change the device conclusions for the solution-deposited and subtractive methods like solution spin coating, compatible with large-scale dense circuit integration.

### All WSe_2_ 1T1R memory cell integration and characterization

The approaches of in-memory and neuromorphic computing based on embedded memory have recently garnered great momentum, with growing interests to apply ReRAM^55^. However, high-density cross-bar ReRAM array suffers from current cross-talk interference due to sneak currents^56^, resulting in misreading and unintended disturbance of memory states as well as undesirable increase in memory standby power consumption. By employing a select transistor to isolate the selected ReRAM cell from unselected cells, a 1T1R architecture can be implemented to circumvent these problems^57,58^. Since the 1T1R cell leakage is gated by the select transistor off-state leakage, it is necessary for the select transistor bandgap to be appropriately wide to limit S/D band-to-band leakage current due to the memory operating voltage. WSe_2_ possess suitable bandgap in the range of 1.2 eV (bulk) to 1.6 eV (monolayer), limiting the transistor minimum off-state leakage to be in the order of pA μm^−1^ for operating voltages in the range of 0.8–1.5 V. On the other hand, the maximum on-state drive current of the select transistor should support the set voltage and reset current of the ReRAM. However, the low intrinsic drive current of 2D TMD-based TFT makes it difficult to drive the ReRAM. Therefore, we propose to utilize the performance enhancement in plasma-oxidized WSe_2_ to mitigate this issue.

**Fig. 4:**
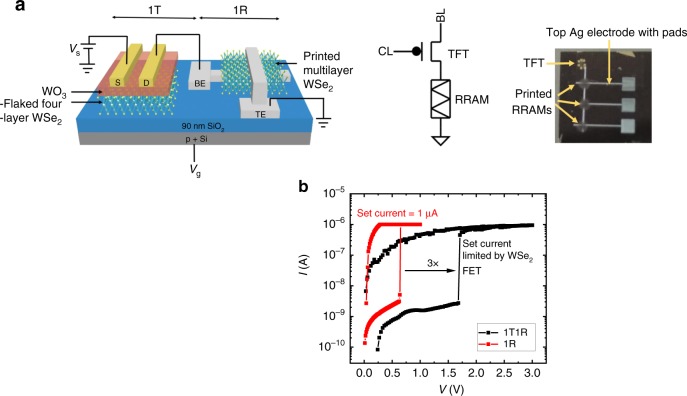
1T1R configuration and characterization. **a** 3D schematic view of 1T1R structure with flaked WSe_2_ transistor and printed WSe_2_ ReRAM and the corresponding circuit representation. The photo image of 3× WSe_2_ ReRAMs printed using low-temperature aerosol jet printing method, which is linked up to the fabricated WSe_2_ TFT on a single chip is also shown. **b** *I*–*V* switching plot for 1T1R configuration, where the switching current is limited by the transistor drive current

We integrated the TFT and the ReRAM on the same chip to study the co-integration and its functionality (Fig. 4a), where the WSe_2_ ReRAM is printed after the WSe_2_ TFT fabrication. The measured 1T1R circuit configuration is as depicted in Fig. 4a. Figure 4b shows the successful switching of the WSe_2_ ReRAM by the WSe_2_ TFT. As expected, the TFT’s on-state resistance increased the memory cell switching voltage to 1.7 V, which is almost 3× larger than that of the ReRAM alone. This clearly highlights the gating impact of the select transistor performance for the memory cell. It is necessary to decrease the TFT on-state resistance while maintaining low off-state leakage to limit sneak current. This becomes increasingly challenging with decreasing cell size where TFT area is constrained. In the next section, we investigate the cell design with the use of material-calibrated compact models and circuit simulations, which would allow us to project for scaled-up memory array implementation.

### Material-device-circuit co-design of 1T1R memory cell

**Fig. 5:**
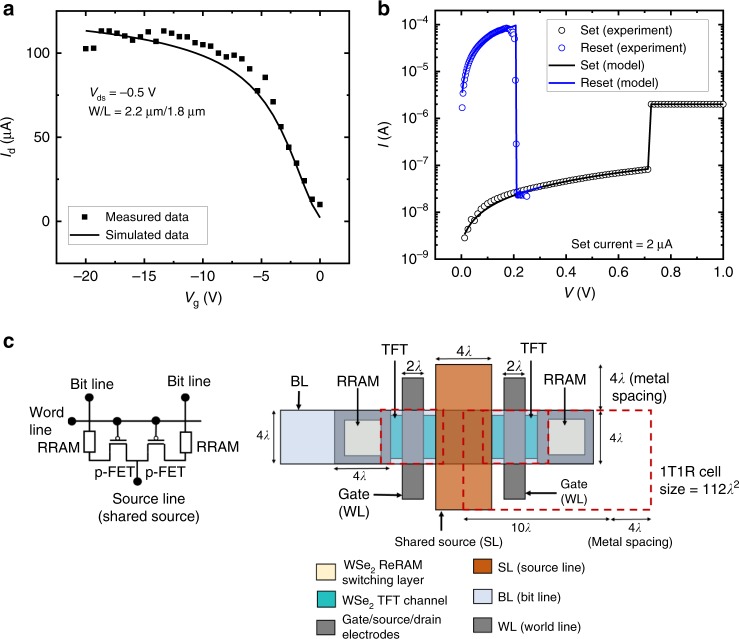
Compact modeling and circuit simulations. **a** *I*_d_*–V*_g_ of SPICE TFT model vs. measured WSe_2_ *p*-FET. **b** *I*–*V* of SPICE ReRAM model vs. measured for SET and RESET process. **c** Circuit representation and layout of shared SL 1T1R structure, with the 1T1R cell size indicated (BL—bit line; WL—word line; SL—shared-source line)

In order to evaluate the memory cell for scaled technologies and to project for future 1T1R technology, we investigate the material-system co-design using detailed circuit modeling and study the disruptive impact of material properties on the system design considerations. A BSIM-IMG compact circuit model^59^ description of the TFT has been calibrated to experiment-based long-channel devices and known WSe_2_ material parameters. Short-channel effects such as velocity saturation, GISL (gate-induced source leakage) and GIDL (gate-induced drain leakage) has been taken into account for the scaled devices through modeling. A hysteron-based compact model, as reported by Garcia-Redondo et al.^60^, has been calibrated to the WSe_2_ ReRAM. Guided by experimental data, we applied these models largely behaviorally, given that the physics of these devices are not well described yet. Despite this, we expect these models to be accurate for our SPICE circuit analysis. Figure 5a, b show the compact model behavior for WSe_2_ TFT and ReRAM, respectively, which correlates well with the experimental data.

**Fig. 6:**
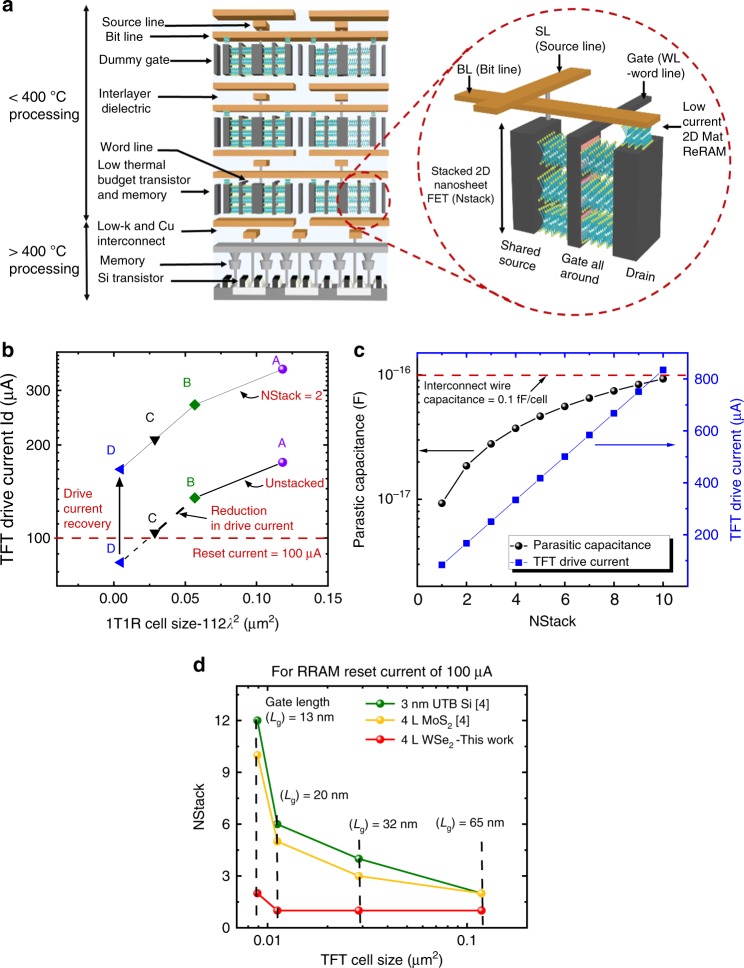
3D monolithic stacking of TFT and memory. **a** Conceptual illustration of 3D monolithic stacking of CMOS logic and 2D multiple-stacked WSe_2_ TFTs with ReRAM with thermal budget indicated for various levels (not to scale). **b** Transistor drive current (at *V*_sg_ = 2 V) variation with respect to 1T1R cell size as per *λ* design rule. The specifications for the legends are, A: *L*_g_ *=* 65 nm*, W*_ch_ *=* 130 nm, EOT = 2.3 nm, *k* = 4.5*;* B: *L*_g_ = 45 nm, *W*_ch_ = 90 nm, EOT = 2.2 nm, *k* = 4.5; C: *L*_g_ = 32 nm, *W*_ch_ = 64 nm, EOT = 2.1 nm, *k* = 4.5; D: *L*_g_ = 13 nm, *W*_ch_ = 26 nm, EOT = 1.1 nm, *k* = 25. We observe ~2.25× drop in drive current as the width is scaled 5× (130–26 nm). Stacked-channel devices showing the recovery of drain current with NStack = 2, to support the ReRAM reset current of 100 µA. **c** Change in TFT drive current and parasitic capacitance (self-capacitance due to stacking) vs. NStack (number of 2DMat nanosheet stacking layer) at *V*_sg_ = 2 V. **d** Comparison of NStack (number of nanosheet TFT stacked) for different feature sizes among WSe_2_, MoS_2_, and UTB Si to support ReRAM reset current of 100 µA

We project scaled technology performance by calibrating our device and circuit models with intrinsic long-channel mobility and contact resistance enhancement salient to our 2D WSe_2_ approach. Our aim is to provide a first-order comparison between different material systems and their potential system impact, without the distraction of subjective details specific to scaled device design (such as interface trap density, S/D tunneling, etc. as explained in Supplementary Fig. 12 and Supplementary Discussion 2) and other more complex technology factors. We recognize that detailed technology factors related to scaled transistor/memory device behavior, interconnect properties, physical layout, and process integration approaches would be useful to refine the system view in the future. Here, we analyze the 1T1R cell scaling using *λ*-based design rule description, where *F* = 4*λ* = minimum metal ½ pitch and 1T1R cell size is limited by the select transistor size (min. cell area = 112*λ*^2^)^61^. The layout of such a shared-source 1T1R cell is shown in Fig. 5c. As the 1T1R memory cell is scaled down, the selector drive current degrades with the linear reduction of width (*W* *=* *4λ*), whereas the ReRAM switching current is largely insensitive to the cell size due to filamentary switching^43^. This would raise concern over the ability of the select transistor to set and reset the ReRAM, for smaller cells. For shorter channel length, as the drive current scales with width (*W*) and *C*_ox_ as per the relation, *I*_sd,sat_ = *V*_sat_*WC*_ox_(*V*_sg_ *−* |*V*_tp_| − *V*_sd,sat_), increasing TFT gate capacitance (*C*_ox_), with thinner high-*k* gate dielectrics (to increase carrier charge density) may compensate for the current degradation due to width scaling^62^. However, our analysis shows that, even with aggressive scaling of high-*k* gate oxide as per industry standards for low-power devices^62,63^, we would still suffer a 2.2× drop in current as the width is reduced 5× (from gate length of 65 to 13 nm in Fig. 6b).

One way to address the issue of weak select transistor would be to rely on smaller ReRAM set current, but at the expense of reduced HRS/LRS ratio (Supplementary Fig. 13). Hence, to mitigate the drive current degradation, without compromising on the memory window, we propose increasing the effective width by vertical stacking of 2DMat nanosheet TFT channels. This would allow for TFT drive current recovery, without sacrificing the 1T1R cell footprint. The conceptual representation of such a 3D monolithic stacking of CMOS logic and 2D multiple-stacked WSe_2_ TFTs is shown in Fig. 6a. While thin WO_3_ is still utilized as hole doping layer, an additional gate dielectric with a metal gate wrapped around the nanosheet could be employed, to realize the proposed gate all around (GAA) vertically stacked WSe_2_ TFT. Accordingly, as shown in Fig. 6b, an NStack (number of 2DMat nanosheet TFT channels) of 2 would more than compensate for the drive current loss due to geometric width scaling, to support ReRAM reset current of 100 µA.

Although stacking of channel layers would result in boosted drive current per footprint, the parasitic capacitance arising from the self-capacitance due to stacking including gate to S/D capacitance and other fringing components could lead to increased switching delays and slowing down of circuit operation. Hence, it is necessary to evaluate the trade-off between the number of stacking layers and the cell switching delay due to transistor capacitance vs. wiring interconnect parasitic. The increase in parasitic capacitance with the number of stacking layers for *L*_g_ = 13 nm is shown in Fig. 6c. With a metal wire pitch of 52 nm and assuming aspect ratio to be 2, the capacitance of the wire is 1.045 fF µm^−1^ ^64^. Accordingly, the interconnect capacitance for a 1T1R cell, considering the metal line length to be 14*λ*, is 0.1 fF per cell. The simulated transistor parasitic capacitance due to stacking reveals that the interconnect capacitance induced by long word line/bit line would be the more dominant factor and that the stacking-induced self-capacitance is not expected to pose a serious concern for NStack ≤10. The necessity for having a high performant stacking channel layer becomes even more critical, to restrict the NStack below 10. With GAA nanosheet FETs being regarded as a potential candidate for sub-3 nm technology node, the key research areas that require improvements are fine-tuning of nanosheet width optimization with extreme ultraviolet lithography^65^, optimization of inner spacers^66^, advancement in metrology, and inspection to measure the buried channel, process control, and other fabrication challenges in gate stack integration.

Furthermore, we have compared the number of stacking layers that would be required for other 2D materials such as MoS_2_ as well as conventional ultrathin Si from other reported works with respect to the WSe_2_ device reported in this work. Our analysis shows that the enhanced WSe_2_ device requires a fewer number of channel stacking layers as compared to other materials, to support the maximum reset current of 100 µA of our low-voltage ReRAM (Fig. 6d). This is due to WSe_2_’s higher mobility at sub-5 nm channel thickness, compared to MoS_2_ and ultrathin Si. Specifically, only two 2D WSe_2_ TFT channel stacks (NStack = 2) are required for cell sizes below 0.01 µm^2^. These findings imply that, apart from thermal budget limitation, the large number of stacking layers required for ultrathin Si transistors and MoS_2_ at sub-5 nm channel thickness increases the complexity of fabrication and the stacking-induced parasitic capacitance.

While smaller effective mass (m*) of WSe_2_ allows for higher mobility and high performance in sub-10 nm gate length (*L*_g_), the enhanced S/D tunneling due to lower m* is the down side^67,68^. Hence, to further reduce the footprint of each device, we recommend greater width scaling rather than *L*_g_ scaling, without increasing the standby power. However, the width scaling will come at the expense of lower drive current per TFT. In this case, channel stacking of TFT becomes even more necessary to recover the required drive current and is an essential control knob to enable dense 1T1R cell.

## Discussion

In this work, a low-thermal-budget hybrid (solution-processed-exfoliated) integration of 2D material-based 1T1R is demonstrated for the first time. We highlight the importance of different material morphology for logic and memory operation. The select transistor needs to be single crystalline with enhanced drive for scaled 1T1R cells, while it is desired for the memory device to be polycrystalline with defects that enable low-voltage switching. We show by post-contact plasma oxidation, a simple low-thermal-budget method to enhance the multilayer WSe_2_ transistor for this purpose; achieving significant hole mobility (230 cm^2^ V^−1^ s^−1^), reduction of contact resistance (to 4.3 kΩµm) and Schottky barrier (to 25 meV). This culminates in a 100× drive enhancement with respect to our control devices. In addition, we report an all-printed WSe_2_-based ReRAM using a low-temperature, aerosol jet process. The ReRAM exhibits sub-1 V non-volatile unipolar switching with a low switching energy of 2.9 pJ per bit. We demonstrated the TFT-ReRAM 1T1R hybrid co-integration, which guided our accurate device-circuit models and enabled us to investigate material-system memory cell co-design for scaled technologies. This led us to the proposed stacked TFT channels for the memory cell to achieve high-density 1T1R memory array for future dense monolithic 3D memory systems.

### Methods

#### Remote plasma oxidation

The plasma chamber source to sample distance is limited to 10 cm. The oxidation is performed at room temperature with a plasma power of 11 W and chamber pressure of 20 mTorr (100 sccm of O_2_ and 20 sccm of Ar) for 2 min to form 2.2 nm WO_3_. For some of the material characterization, the WO_3_ is removed by dipping in 1 M KOH solution for 30 s.

#### TFT device fabrication and characterization

WSe_2_ flakes were mechanically exfoliated on p + Si with 90 nm SiO_2_ layer, followed by electrode patterning using electron beam lithography. The length and width of the device are characterized and validated by AFM. Ag (10 nm) contacts capped with Au (90 nm) was deposited by electron beam evaporator followed by lift off to form source and drain contacts. The device is then subjected to an annealing procedure (200 °C for 1 h in N_2_-H_2_ ambient, followed by vacuum annealing at 250 °C for 0.5 h), to remove the photoresist residue and other gaseous adsorbates. After which, the plasma oxidation, as explained in the previous step is performed to form WO_3_ above the channel and also underneath the S/D contacts. The electrical measurements were collected by Agilent parameter analyzer B1500A.

#### Aerosol ink printing method for WSe_2_ ReRAM

WSe_2_ flakes suspended in ethanol forms the ink (concentration 0.1 mg/ml, from 2D semiconductor) that is ultrasonically atomized and deposited by the Optomec AJ5X Aerosol Jet 5-axis Printer^69^. The bottom and top Ag electrodes are printed via the pneumatic atomizer followed by an 830 nm laser sintering process (Kapton) or a 150 °C, 30 min baking process (SiO_2_/Si substrate). Due to the low concentration of WSe_2_ in the ink, the ReRAM WSe_2_ layer has to be deposited over multiple passes. We deposited ~400 nm average thickness of WSe_2_ (printed over 30 passes) for our ReRAM devices, with more details provided in the Supplementary Information (Supplementary Table 3 and Supplementary Fig. 8a). A final step of baking the entire sample at a temperature of 100 °C for 30 min is done to ensure conductivity of the printed Ag electrodes.

## Supplementary information

Supplementary Information

## Data Availability

All data supporting the findings of this study are available from the corresponding author on request.

## References

[1] Shulaker, M. M. et al. Monolithic 3D integration: a path from concept to reality. In *Proc. 2015 Design, Automation & Test in Europe Conference & Exhibition* 1197–1202 (EDA Consortium, 2015).

[2] Fox, R. et al. High performance *k* = 2.5 ULK backend solution using an improved TFHM architecture, extendible to the 45nm technology node. In *Electron Devices Meeting, 2005. IEDM Technical Digest. IEEE International* 81–84 (IEEE, 2005).

[3] Batude, P. et al. 3D sequential integration opportunities and technology optimization. In *IEEE International Interconnect Technology Conference* 373–376 (IEEE, 2014).

[4] Iannaccone G, Bonaccorso F, Colombo L, Fiori G (2018). Quantum engineering of transistors based on 2D materials heterostructures. Nat. Nanotechnol..

[5] Fu Y, Qin Y, Wang T, Chen S, Liu J (2010). Ultrafast transfer of metal-enhanced carbon nanotubes at low temperature for large-scale electronics assembly. Adv. Mater..

[6] Castellanos-Gomez A (2014). Deterministic transfer of two-dimensional materials by all-dry viscoelastic stamping. 2D Mater..

[7] Wang, C.-H. et al. 3D monolithic stacked 1T1R cells using monolayer MoS_2_ FET and hBN RRAM fabricated at low (150 °C) temperature. In *2018 IEEE International Electron Devices Meeting (IEDM)* 22–25 (IEEE, 2018).

[8] Batude, P. et al. 3D sequential integration: application-driven technological achievements and guidelines. In *2017 IEEE International Electron Devices Meeting (IEDM)* 1–3 (IEEE, 2017).

[9] Akinwande D, Petrone N, Hone J (2014). Two-dimensional flexible nanoelectronics. Nat. Commun..

[10] Fang H (2012). High-performance single layered WSe_2_ p-FETs with chemically doped contacts. Nano Lett..

[11] Resta GV (2016). Polarity control in WSe_2_ double-gate transistors. Sci. Rep..

[12] Das S, Dubey M, Roelofs A (2014). High gain, low noise, fully complementary logic inverter based on bi-layer WSe_2_ field effect transistors. Appl. Phys. Lett..

[13] Pradhan NR (2015). Hall and field-effect mobilities in few layered p-WSe_2_ field-effect transistors. Sci. Rep..

[14] Liu W (2013). Role of metal contacts in designing high-performance monolayer n-type WSe_2_ field effect transistors. Nano Lett..

[15] Shokouh SHH (2015). High-performance, air-stable, top-gate, p-channel WSe_2_ field-effect transistor with fluoropolymer buffer layer. Adv. Funct. Mater..

[16] Chuang H-J (2016). Low-resistance 2D/2D ohmic contacts: a universal approach to high-performance WSe_2_, MoS_2_, and MoSe_2_ transistors. Nano Lett..

[17] Chuang H-J (2014). High mobility WSe_2_ p- and n-type field-effect transistors contacted by highly doped graphene for low-resistance contacts. Nano Lett..

[18] Kappera R (2014). Phase-engineered low-resistance contacts for ultrathin MoS_2_ transistors. Nat. Mater..

[19] Castellanos-Gomez A (2012). Laser-thinning of MoS_2_: on demand generation of a single-layer semiconductor. Nano Lett..

[20] Yamamoto M, Nakaharai S, Ueno K, Tsukagoshi K (2016). Self-limiting oxides on WSe_2_ as controlled surface acceptors and low-resistance hole contacts. Nano Lett..

[21] Pudasaini PR (2018). High-performance multilayer WSe_2_ field-effect transistors with carrier type control. Nano Res..

[22] Zhang R, Drysdale D, Koutsos V, Cheung R (2017). Controlled layer thinning and p‐type doping of WSe_2_ by vapor XeF_2_. Adv. Funct. Mater..

[23] Li Z (2016). Layer control of WSe_2_ via selective surface layer oxidation. ACS Nano.

[24] Liu Y (2015). Thermal oxidation of WSe_2_ nanosheets adhered on SiO_2_/Si substrates. Nano Lett..

[25] Liu B (2016). High-performance WSe_2_ field-effect transistors via controlled formation of in-plane heterojunctions. ACS Nano.

[26] Roy T (2014). Field-effect transistors built from all two-dimensional material components. ACS Nano.

[27] Meyer J (2012). Transition metal oxides for organic electronics: energetics, device physics and applications. Adv. Mater..

[28] Yamamoto M (2015). Self-limiting layer-by-layer oxidation of atomically thin WSe_2_. Nano Lett..

[29] Lee S, Tang A, Aloni S, Philip Wong H-S (2015). Statistical study on the Schottky barrier reduction of tunneling contacts to CVD synthesized MoS_2_. Nano Lett..

[30] Pan C (2017). Coexistence of grain-boundaries-assisted bipolar and threshold resistive switching in multilayer hexagonal boron nitride. Adv. Funct. Mater..

[31] Qian K (2016). Hexagonal boron nitride thin film for flexible resistive memory applications. Adv. Funct. Mater..

[32] Hao C (2016). Liquid-exfoliated black phosphorous nanosheet thin films for flexible resistive random access memory applications. Adv. Funct. Mater..

[33] Son D (2016). Colloidal synthesis of uniform-sized molybdenum disulfide nanosheets for wafer-scale flexible nonvolatile memory. Adv. Mater..

[34] Tan C, Liu Z, Huang W, Zhang H (2015). Non-volatile resistive memory devices based on solution-processed ultrathin two-dimensional nanomaterials. Chem. Soc. Rev..

[35] Bessonov AA (2015). Layered memristive and memcapacitive switches for printable electronics. Nat. Mater..

[36] Cheng P, Sun K, Hu YH (2015). Memristive behavior and ideal memristor of 1T phase MoS_2_ nanosheets. Nano Lett..

[37] Sangwan VK (2015). Gate-tunable memristive phenomena mediated by grain boundaries in single-layer MoS_2_. Nat. Nanotechnol..

[38] Zhang F (2019). Electric-field induced structural transition in vertical MoTe_2_ and Mo_1–*x*_W_*x*_Te_2_-based resistive memories. Nat. Mater..

[39] Tao H (2017). Scalable exfoliation and dispersion of two-dimensional materials—an update. Phys. Chem. Chem. Phys..

[40] Tsai T-L, Chang H-Y, Jiang F-S, Tseng T-Y (2015). Impact of post-oxide deposition annealing on resistive switching in HfO_2_-based oxide RRAM and conductive-bridge RAM devices. IEEE Electron Device Lett..

[41] Wang M (2018). Robust memristors based on layered two-dimensional materials. Nat. Electron..

[42] Strukov DB, Alibart F, Williams RS (2012). Thermophoresis/diffusion as a plausible mechanism for unipolar resistive switching in metal–oxide–metal memristors. Appl. Phys. A.

[43] Ge R (2017). Atomristor: nonvolatile resistance switching in atomic sheets of transition metal dichalcogenides. Nano Lett..

[44] Luo Q (2016). Super non-linear RRAM with ultra-low power for 3D vertical nano-crossbar arrays. Nanoscale.

[45] Raghavan N (2014). Performance and reliability trade-offs for high-κ RRAM. Microelectron. Reliab..

[46] Cheng L (2017). Reprogrammable logic in memristive crossbar for in-memory computing. J. Phys. D.

[47] Breuer T (2016). Realization of minimum and maximum gate function in Ta_2_O_5_-based memristive devices. Sci. Rep..

[48] Huang P (2016). Reconfigurable nonvolatile logic operations in resistance switching crossbar array for large-scale circuits. Adv. Mater..

[49] Yan X (2017). Highly improved performance in Zr_0.5_Hf_0.5_O_2_ films inserted with graphene oxide quantum dots layer for resistive switching non-volatile memory. J. Mater. Chem. C.

[50] Lee S, Sohn J, Jiang Z, Chen H-Y, Wong H-SP (2015). Metal oxide-resistive memory using graphene-edge electrodes. Nat. Commun..

[51] Govoreanu, B. et al. 10 × 10 nm^2^ Hf/HfO_*x*_ crossbar resistive RAM with excellent performance, reliability and low-energy operation. In *Electron Devices Meeting (IEDM), 2011 IEEE International* 31–36 (IEEE, 2011).

[52] Zhao X (2017). Confining cation injection to enhance CBRAM performance by nanopore graphene layer. Small.

[53] Shih C-C (2016). Ultra-low switching voltage induced by inserting SiO_2_ layer in indium–tin–oxide-based resistance random access memory. IEEE Electron Dev. Lett..

[54] Song YL (2011). Low reset current in stacked AlO_*x*_/WO_*x*_ resistive switching memory. IEEE Electron Dev. Lett..

[55] Ielmini D, Wong H-SP (2018). In-memory computing with resistive switching devices. Nat. Electron..

[56] Zhou J, Kim K-H, Lu W (2014). Crossbar RRAM arrays: selector device requirements during read operation. IEEE Trans. Electron Dev..

[57] Wang, X. P. et al. Highly compact 1T-1R architecture (4F^2^ footprint) involving fully CMOS compatible vertical GAA nano-pillar transistors and oxide-based RRAM cells exhibiting excellent NVM properties and ultra-low power operation. In *Electron Devices Meeting (IEDM), 2012 IEEE International* 20–26 (IEEE, 2012).

[58] Chen P-Y, Yu S (2015). Compact modeling of RRAM devices and its applications in 1T1R and 1S1R array design. IEEE Trans. Electron Dev..

[59] Chauhan, Y. S. et al. BSIM compact MOSFET models for SPICE simulation. In *Mixed Design of Integrated Circuits and Systems (MIXDES)*, *2013 Proceedings of the 20th International Conference* 23–28 (IEEE, 2013).

[60] Garcia-Redondo F, Gowers RP, Crespo-Yepes A, López-Vallejo M, Jiang L (2016). SPICE compact modeling of bipolar/unipolar memristor switching governed by electrical thresholds. IEEE Trans. Circuits Syst. I.

[61] Yeh C-WS, Wong SS (2015). Compact one-transistor-N-RRAM array architecture for advanced CMOS technology. IEEE J. Solid-State Circuits.

[62] Robertson J (2005). High dielectric constant gate oxides for metal oxide Si transistors. Rep. Prog. Phys..

[63] Lee BH, Oh J, Tseng HH, Jammy R, Huff H (2006). Gate stack technology for nanoscale devices. Mater. Today.

[64] Li H (2017). Device and circuit interaction analysis of stochastic behaviors in cross-point RRAM aArrays. IEEE Trans. Electron Dev..

[65] Barraud, S. et al. Performance and design considerations for gate-all-around stacked-nanowires FETs. In *2017 IEEE International Electron Devices Meeting (IEDM)* 22–29 (IEEE, 2017).

[66] Huang, Y.-C., Chiang, M.-H. & Wang, S.-J. Speed optimization of vertically stacked gate-all-around MOSFETs with inner spacers for low power and ultra-low power applications. In *20th International Symposium on Quality Electronic Design (ISQED)* 231–234 (IEEE, 2019).

[67] Majumdar K, Hobbs C, Kirsch PD (2014). Benchmarking transition metal dichalcogenide MOSFET in the ultimate physical scaling limit. IEEE Electron Dev. Lett..

[68] Cao W, Kang J, Sarkar D, Liu W, Banerjee K (2015). 2D semiconductor FETs—projections and design for sub-10 nm VLSI. IEEE Trans. Electron Dev..

[69] Secor Ethan B (2018). Principles of aerosol jet printing. Flexible and Printed Electronics.

