# Supplementary material for: All WSe2 1T1R resistive RAM cell for future monolithic 3D embedded memory integration
Source: Nat Commun. 2019 Nov 15;10:5201. doi: 10.1038/s41467-019-13176-4 (PMC6858359; doi:10.1038/s41467-019-13176-4)
Supplement: Supplementary file 1 — Supplementary Information [file 41467_2019_13176_MOESM1_ESM.pdf]

Supplementary Information

All WSe_2 1T1R Resistive RAM Cell for Future Monolithic 3D Embedded Memory Integration

Maheswari Sivan, Yida Li*, Hasita Veluri, Yunshan Zhao, Tang Baoshan, Xinghua Wang, Evgeny Zamburg, Jin Feng Leong, Jessie Xuhua Niu, Umesh Chand, and Aaron Voon-Yew Thean*

Department of Electrical and Computer Engineering, National University of Singapore, 4 Engineering Drive 3,

Singapore 117583

Email contacts: li.yida@nus.edu.sg,Aaron.Thean@nus.edu.sg

Supplementary Figures

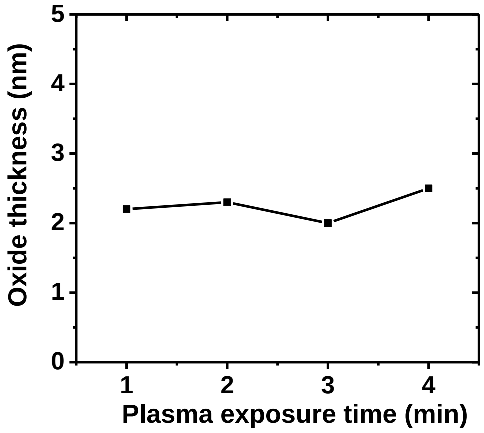

Supplementary Figure 1. Self-limiting Remote Plasma Oxidation. Plot between oxide thickness and plasma exposure time, confirming the self-limiting nature of the oxidation process. The WO_3 thickness remained independent of plasma exposure time.

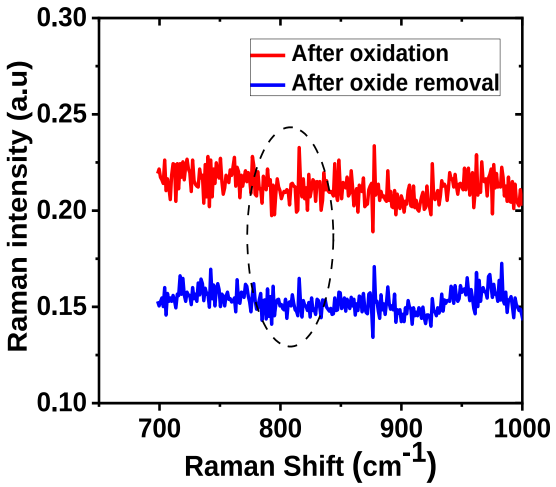

Supplementary Figure 2. Comparison of Raman Spectroscopy after oxidation and after oxide removal. The WO_3 Raman peak is expected to appear at a Raman shift of 800 cm^{-1}(circled part) [1]. However, the absence of such a peak suggests the amorphous nature of WO_3.

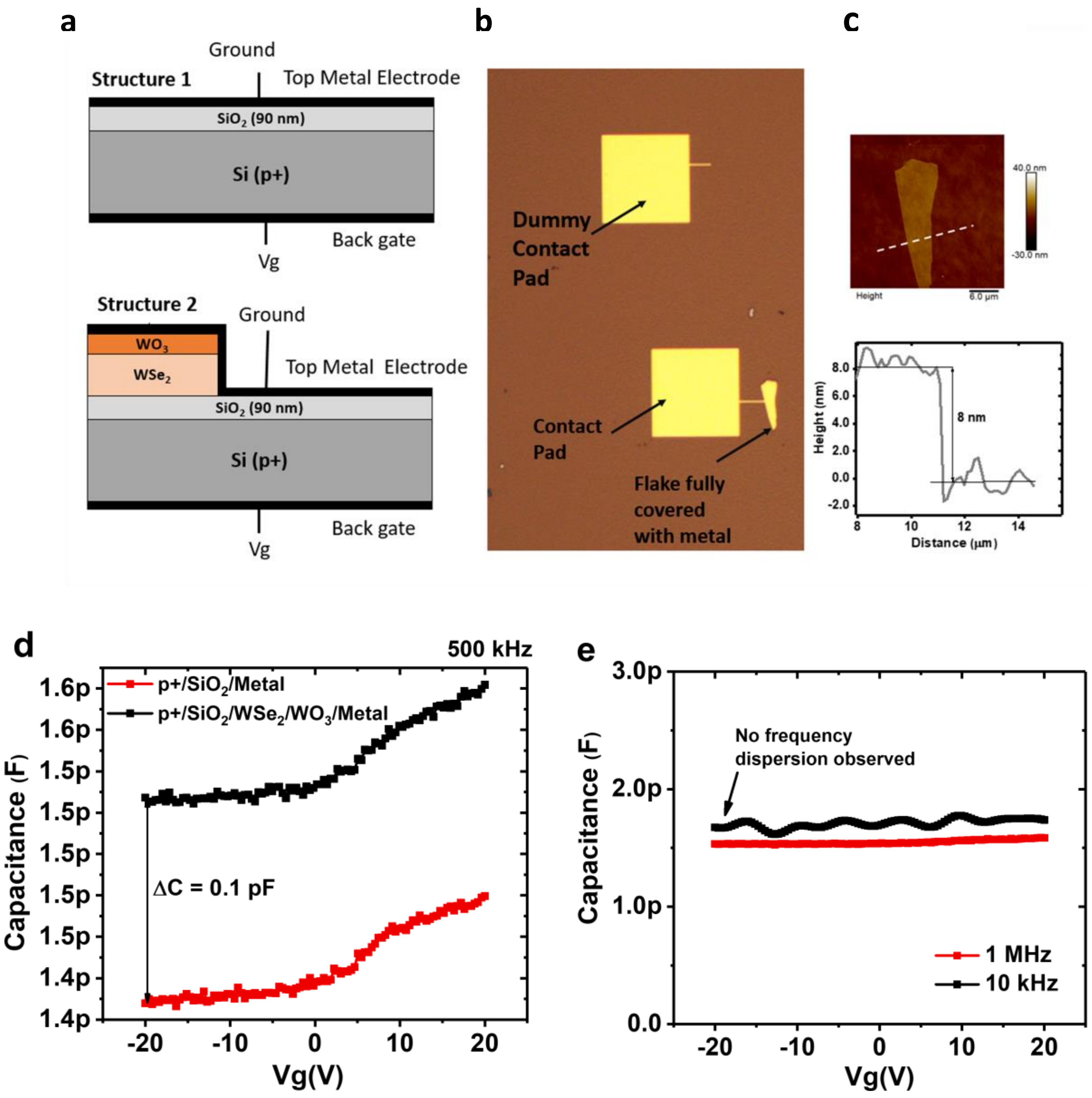

Supplementary Figure 3. Experimental determination of gate oxide capacitance for WSe_2 TFT with WO_3. **a** Schematic representation of p+ Si/SiO_2/Metal and p+ Si/SiO_2/WSe_2/WO_3/Metal stack. **b** Microscope image of dummy contact pad and WSe_2 flake fully covered with metal. The contact area of the WSe_2 flake is 130 μm^2. **c** The AFM height scan of the WSe_2 flake. **d** Capacitance plot of structure 1 and structure 2. **e** Frequency dependent CV plot reveals small (9%) frequency dispersion, ruling out the possibility of any significant spurious charges.

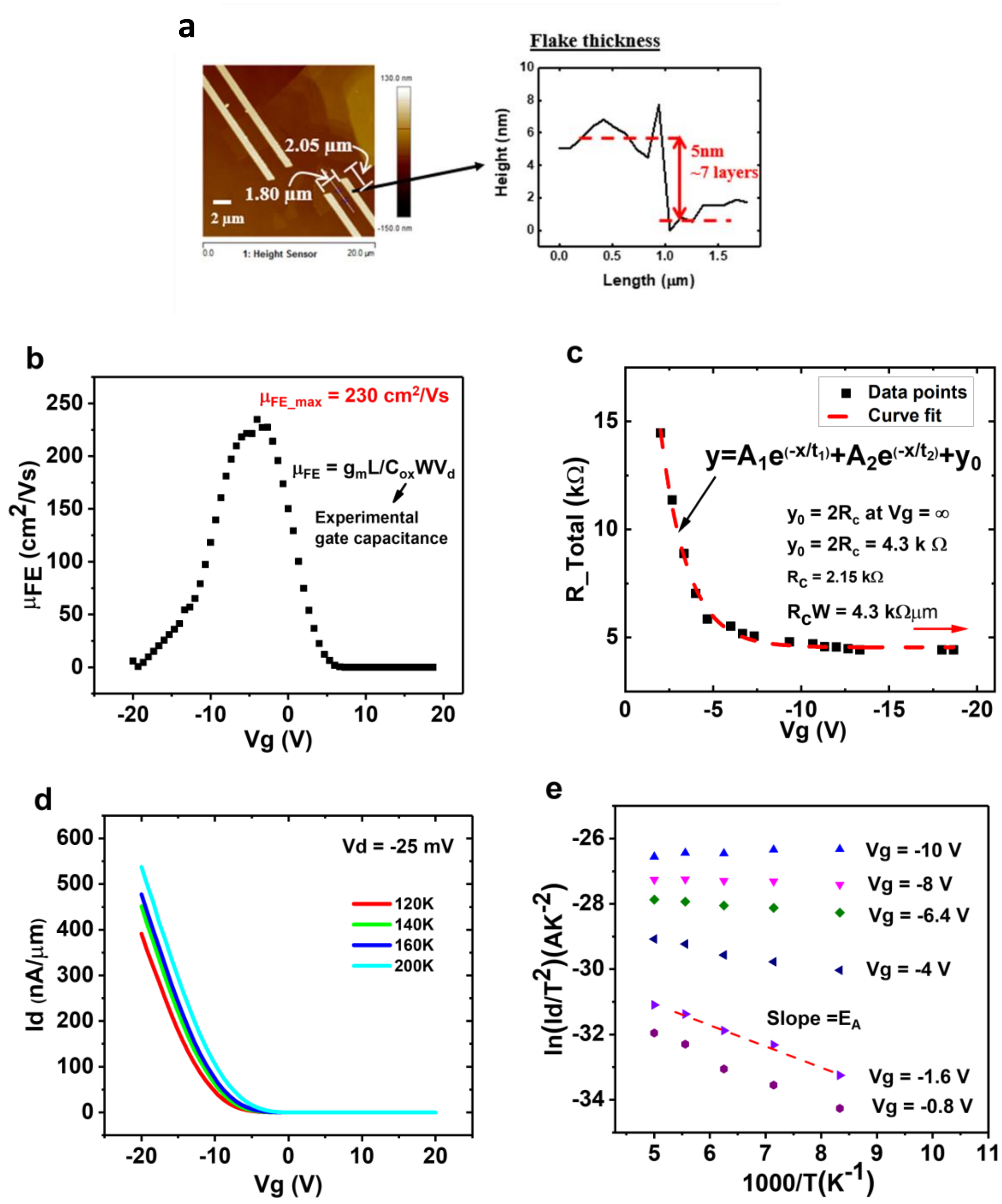

Supplementary Figure 4. Electrical Characterization of post contact plasma oxidized WSe_2 TFT. **a** AFM image of WSe_2 FET after plasma oxidation with thickness indicated. **b** Mobility extraction for the 4 layer WSe_2 TFT with oxide, from maximum transconductance. **c** Contact resistance extracted from R_Total vs V_g curve. R_total becomes $2R_c$ when V_g tends to infinity. **d** Id-Vg for post oxidized WSe_2 TFT for temperatures from 120 K to 200 K. **e** Activation energy extraction from Arrhenius plot.

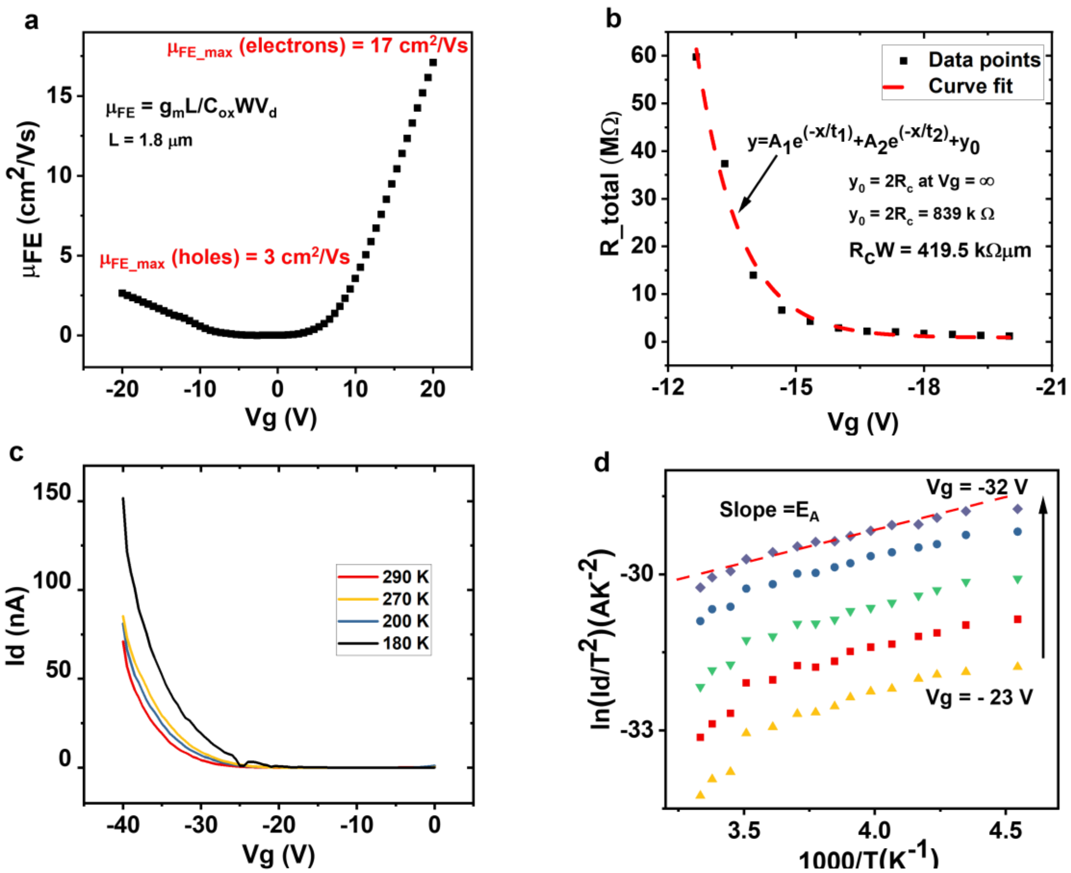

Supplementary Figure 5. Electrical Characterization for 4 layer WSe_2 TFT without oxide. **a** Mobility extraction for the 4 layer WSe_2 TFT without oxide, from maximum transconductance. **b** Contact resistance extracted from R_Total vs V_g curve. R_total becomes $2R_c$ when V_g tends to infinity. **c** Id-Vg for temperatures from 180 K to 290 K at Vd=0.1 V. **d** Activation energy extraction from Arrhenius plot from which Schottky barrier is extracted.

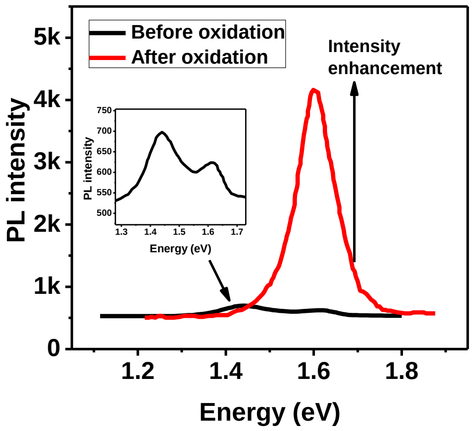

Supplementary Figure 6. Photoluminescence spectroscopy of WSe_2 after plasma oxidation. Comparison of Photoluminescence (PL) spectroscopy before and after oxidation with the same incident PL power is performed. The increase in PL intensity after oxidation due to thinning of WSe_2, rules out the probability of plasma oxidation induced defect creation in the underlying WSe_2.

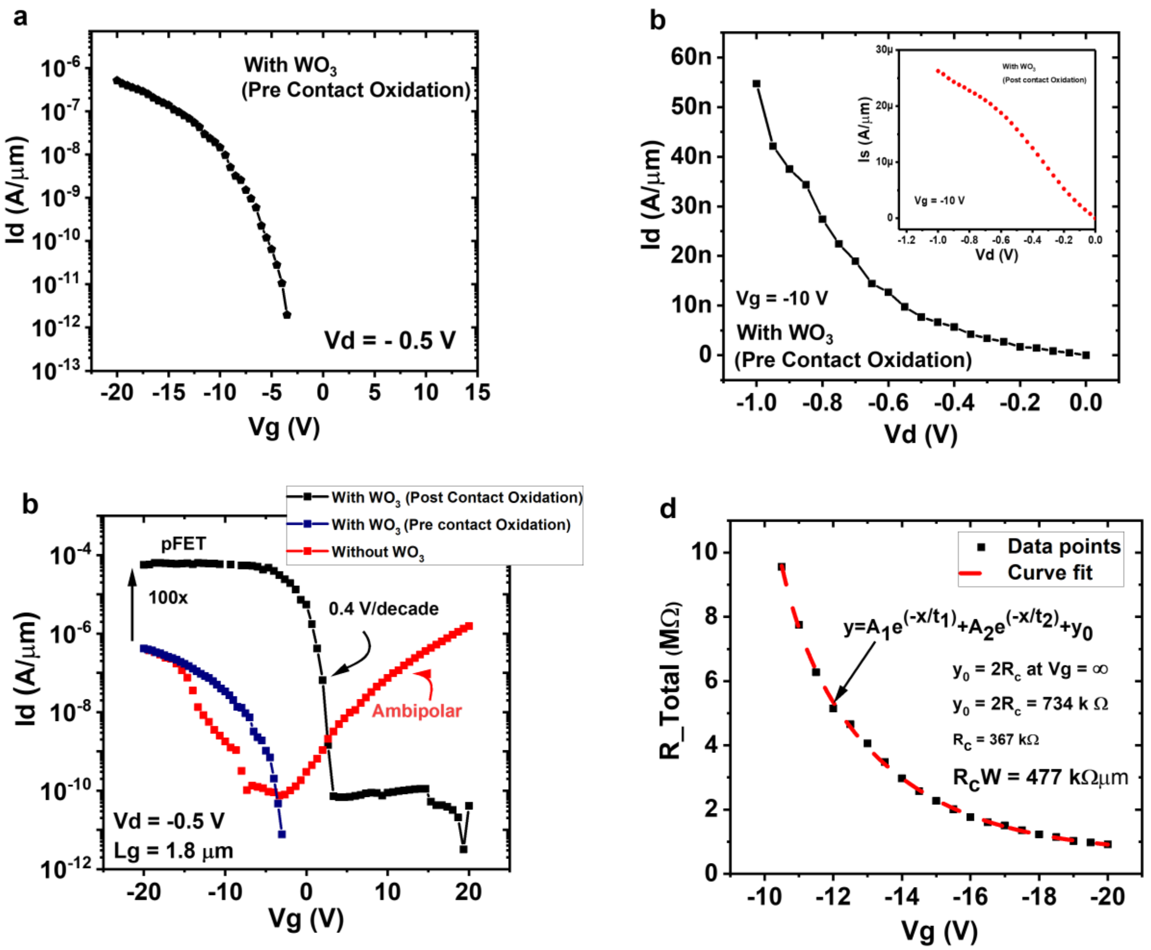

Supplementary Figure 7. Electrical Characterization of Pre-Contact Oxidized WSe_2 TFT. **a** Id-Vg plot of the WSe_2 TFT, where the plasma oxidation step is performed before the metal contact formation (pre-contact oxidation). **b** Output characteristics of the WSe_2 TFT for pre contact oxidation. The behavior is clearly Schottky as opposed to linear output characteristics for the post contact oxidized device, shown in the inset. **c** Transfer characteristic comparison of the pre oxidized device with the post oxidized device. **d** The contact resistance extraction for the pre oxidized device.

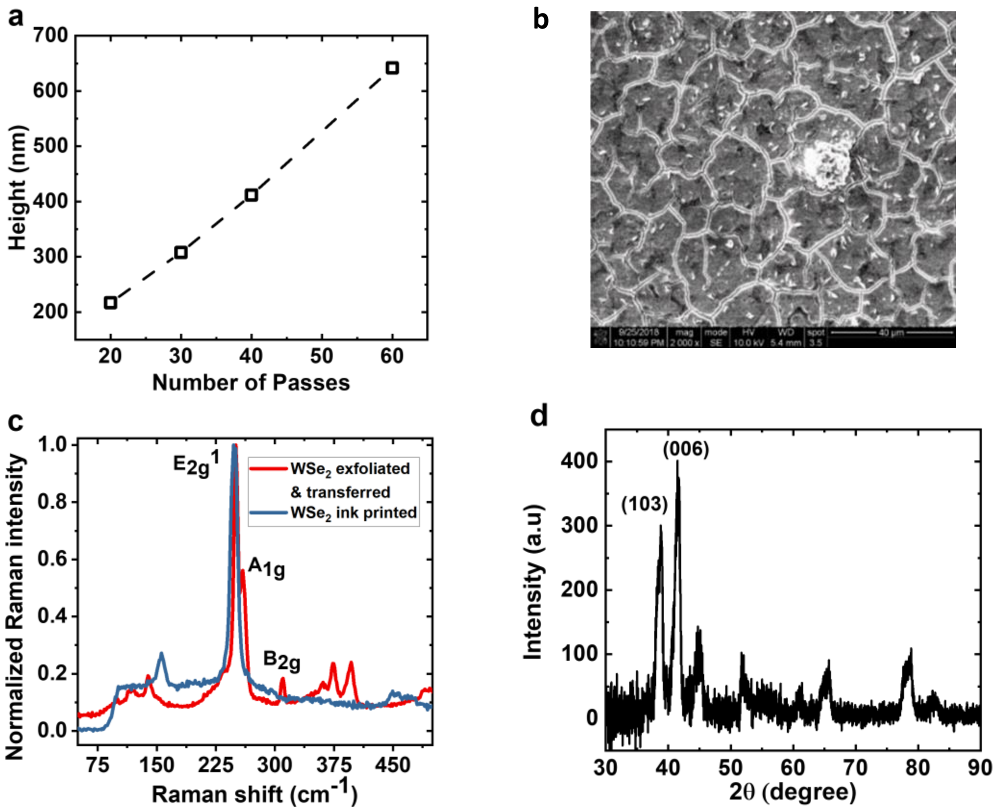

Supplementary Figure 8. Printed WSe_2 ReRAM material characterization. **a** Relationship between the thicknesses of the printed WSe_2 layers and the number of printing passes. **b** SEM image showing the morphology of the printed WSe_2 nano-sheets. **c** Comparison of Raman spectroscopy of mechanically exfoliated few layers WSe_2 and printed WSe_2 from suspended WSe_2 flakes in ethanol solution. **d** XRD spectrum of printed WSe_2.

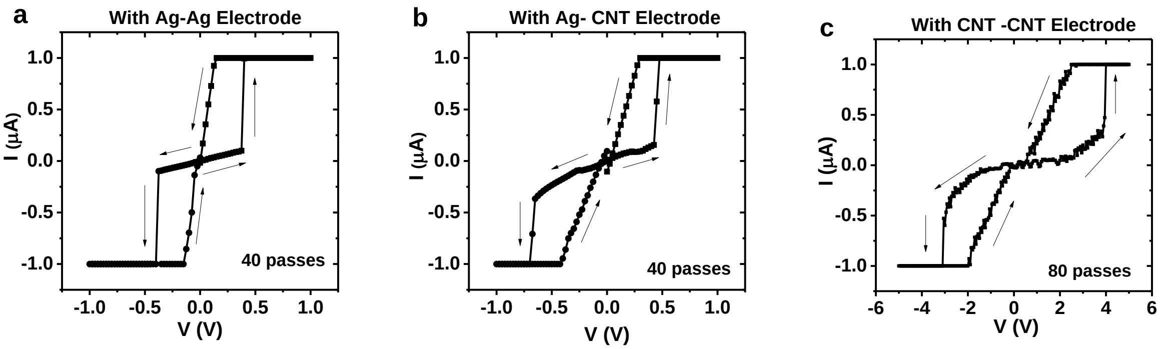

Supplementary Figure 9. Effect of metal electrode on ReRAM switching Characteristics. DC switching sweep for printed WSe_2 ReRAM with **a** Ag-Ag contacts. **b** Ag-CNT contacts. **c** CNT- CNT contacts.

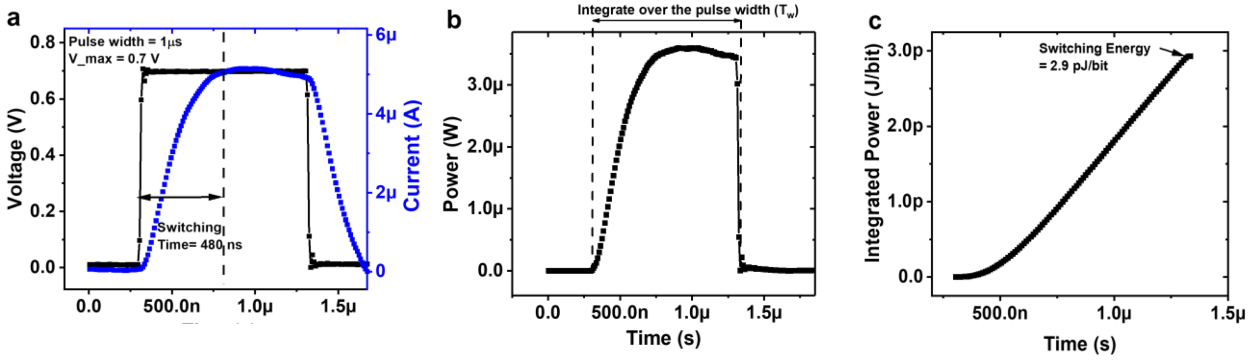

Supplementary Figure 10. ReRAM Switching energy estimation. **a** Switching time characterization with an AC pulse of 0.7V amplitude and 1µs pulse width. **b** Switching power vs time**. c** The Switching power is integrated over the entire pulse width to estimate the switching.

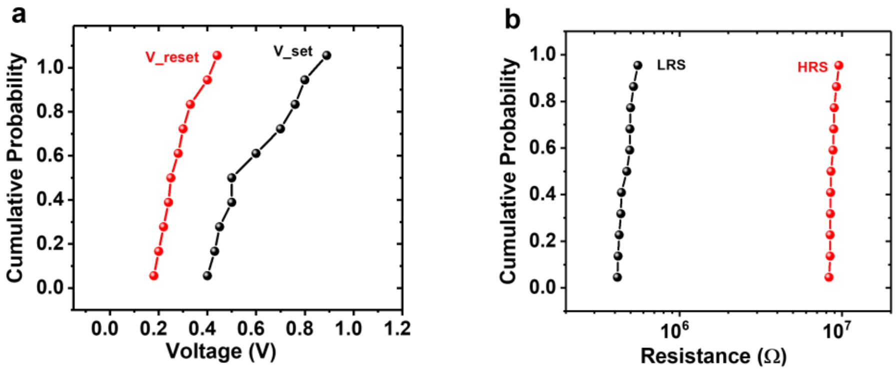

Supplementary Figure 11. Cumulative distribution function plot for 10 representative WSe_2 ReRAM. **a** Cumulative distribution function of set and reset voltage. **b** Cumulative distribution function of LRS and HRS showing small statistical variation

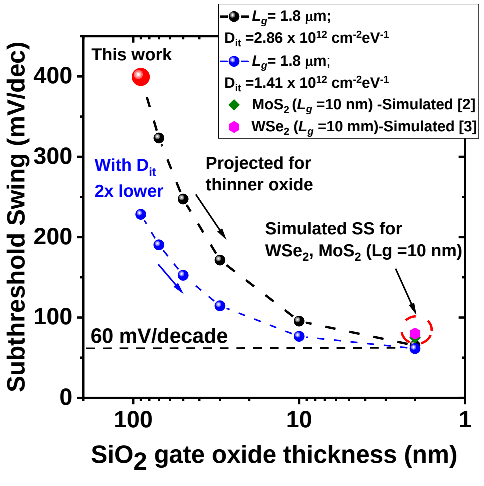

Supplementary Figure 12. Projection of subthreshold swing variation with gate oxide thickness. Subthreshold swing at room temperature (for the 1.8 µm gate length device in the manuscript) is projected against gate oxide thickness and interface trap density (D_{it}). The simulated data points from literature for MoS_2[2] and WSe_2[3] at gate length of 10 nm is also added for reference.

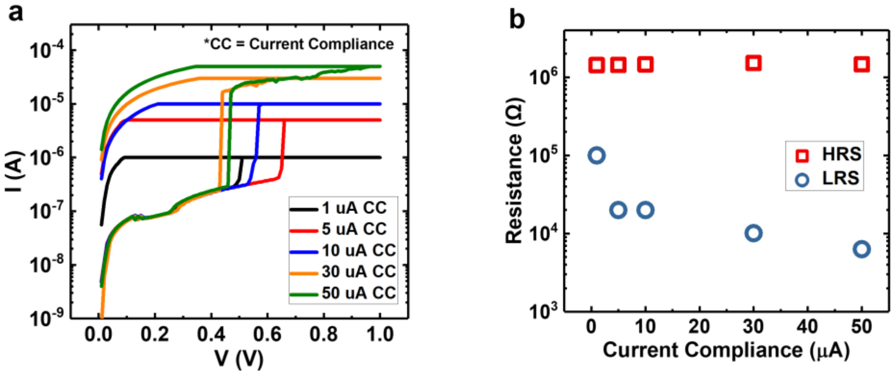

Supplementary Figure 13. Effect of Current Compliance on Memory Window**. a** Multilevel switching characteristics of the printed WSe_2 ReRAM with different current compliance level set. **b** Plot of HRS and LRS with corresponding different current compliance (CC) level applied. As CC increases, and larger low-to-high resistive memory window is obtained. V_{read} of the resistance state is done at 0.1 V.

Supplementary Table

	**Silicon**	**Germanium**	**Carbon Nano Tubes**	**2D Materials (MoS_2, WSe_2, Black Phosphorous)**	**Oxide Semiconductors (ZnO, InGaZnO, NiO)**
**Dopant Activation Temperature**	800-1000°C (*n* & *p*-type)	<400°C (*n* & *p*-type)	N/A (Room Temperature)	N/A (Room Temperature) (*n* & *p*-type)	N/A (Room Temperature) (Mostly *n*-type)
**Effective Mobility ($cm^2V^{-1}s^{-1}$)**	1400 (electron), 260 (holes)	400-600's[4]	20-1000's[5]	10-1000's[6]	10-100's[7]
**Band gap**	1.1 eV	<0.6 eV (Limits V_{dd} <0.6 V)	1-1.3 eV	1-3eV	Wide bandgap > 3eV
**Monolithic 3D Integration Method**	No easy solution for fine-grain transfer & bond at sub-100nm resolution	Epitaxial Growth on Si template, transfer & bound[8]	CVD Growth, Transfer & Bond (Large-scale demonstrated)[9]	CVD Growth, Transfer & Bond[10]	PVD Deposition (Currently 12' fab compatible)[11]

Supplementary Table 1. Choice of materials for 3D monolithic integration. Comparison of different material choices for monolithic 3D integration, with respect to dopant activation temperature and other material characteristics (N/A-not applicable).

Reference	Gate length (µm)	Hole Mobility ($cm^2V^{-1}s^{-1}$)	I_{on} (µA/µm)	I_{off} (pA/ µm)	Contact Resistance (kΩµm)
Yamamoto et al.[12]	5.2	50	0.5	0.01	66
Chuang et al.[13]	0.27	200	50	1000	0.3
Chuang et al.[14]	4.8	204 (at 160 K)	5	0.5	0.2
This work (Without oxide)	1.8	3	0.4	69	477
This work (With oxide)	1.8	230	57	76	4.3

Supplementary Table 2. Performance Benchmark Table for WSe_2 *p*-FET. Comparison of oxidized WSe_2 *p*-FET with other representative reported WSe_2 *p*-FET works.

Process	Parameters
Ag electrode printing	Sheath flow= 60 sccm<br>Carrier flow= 30 sccm
Sintering	Power= 100 mW
WSe_2 layer printing	Sheath flow= 60 sccm<br>Carrier flow= 30 sccm<br>Number of Passes =40

Supplementary Table 3. Aerosol Printing Process parameters. Process parameters used at different stages in the fabrication of printed WSe_2 ReRAM.

Reference	Resistive switching layer	Switching Energy (J/bit)	Memory window
Zhang, F et al.[21]	$MoTe_2$	~18.75 pJ*	10
Lee, Seunghyun, et al.[22]	Graphene/HfO_x/TiN	0.23 pJ	70
Yan, Xiaobing, et al. [23]	$Zr_{0.5}Hf_{0.5}O_2$ –graphene oxide quantum dot	~21 pJ*	10^3
Cheng, Long, et al.[24]	Ti/HfO_2/W	19.5 nJ	10^2
Govoreanu, B et al.[25]	HfO_2	0.1 pJ	50
Shih et al.[26]	SiO_2	~3 nJ*	20
Bessonov, Alexander et al.[16]	MoS_2/MoO_x	~1.5 µJ*	10^4
Song, Y. L et al.[27]	AlO_x/WO_x	~40 pJ*	10^2
Breuer, Thomas, et al. [28]	Ta_2O_5	~3 µJ*	10^3
Huang, Peng, et al.[29]	HfO_x-AlO_y	~0.3 nJ*	10^2
This work	Ag/WSe_2/Ag	2.9 pJ	10^3

Supplementary Table 4. Switching energy vs Memory window. Benchmark Table for switching energy vs memory window with respect to other reported representative oxide based ReRAMs. The symbol * is used to denote that the switching energy is estimated by equation (2).

Reference	Active layer (Thickness)	Switching voltage (V)	Switching Current (A)	Reset voltage (V)	Reset current (A)	Set Power (W)	Reset Power (W)	Switching energy (J/bit)	Endu rance
Son, D. et al.[15]	MoS_2 (Colloidal Synthesis-50 nm)	5.5	0.1 mA	0.5 V	50 mA	550 μW	25 mW	NA	100
Bessono A. A. et al.[16]	MoO_x-MoS_2 (Solution processed-50-100 nm)	0.15	0.5 mA	0.1	1 mA	75 μW	100 μW	100 J/bit	50
Hao, C. et al[17]	Black Phosphorous (Liquid exfoliated-10 nm)	1.5	100 μA	1.5	100 μA	150 μW	150 μW	NA	NA
Huang et al.[18]	Graphene (CVD-2nm)	2	1 μA	0.5	0.5 μA	2 μW	0.2 μW	NA	100
Puglisi et al.[19]	hBN (CVD-5 nm)	0.75	10 mA	0.5	20 mA	7.5 mW	10 mW	NA	100
Xu, Renjing, et al.[20]	MoS_2 (MOCVD-0.7 nm)	0.2	2 mA	0.2	1.5 mA	0.4 mW	0.3 mW	NA	20
This work	Printed WSe_2 (~400 nm)	0.5	2 μA	0.3	40 μA	1 μW	12 μW	2.3 pJ/bit	90

Supplementary Table 5. Performance benchmark for printed Ag/WSe_2/Ag ReRAM. Benchmark Table evaluating performance of printed Ag/WSe_2/Ag ReRAM with respect to other reported two dimensional material based ReRAM. NA-not reported.

Supplementary Notes

Supplementary Note 1: Experimental determination of gate oxide capacitance for WSe_2 TFT with WO_3

Given that the WSe_2 TFT channel area is in the order of tens of μm^2, accurate determination of gate capacitance from the transistor is challenging. Hence we fabricated separate MOS capacitor structures as shown in Supplementary Figure 3a and b. The measurement procedure is explained below:

1. Measure CV for Structure 1 (p+ Si/SiO_2/WSe_2/WO_3/Metal)- M1 (Supplementary Figure 3a)
2. Measure CV for Structure 2 (p+ Si/SiO_2/Metal)- M2 (Supplementary Figure 3a)

Capacitance of (WSe_2+WO_3) =ΔC= M1-M2 (Supplementary Figure 3d)

We found that the experimentally measured capacitance is around 77 nF/cm^2 2x larger than the geometric gate capacitance (38 nF/cm^2)

Supplementary Note 2: ReRAM Switching Energy Estimation

Switching energy per bit, reported in this work is calculated by integrating switching power over the pulse width.

$$E = \int_0^{Tprog} I_{prog}(t)V_{prog}(t)\, dt \qquad (1)$$

Where T_{prog} is the programming voltage pulse width, $V_{prog}(t)$ is the switching voltage and $I_{prog}(t)$ is the switching current.

*For the references, where switching energy is not explicitly stated, we estimated the switching energy (Table at the first order[22] by,

$$E = V_{prog}\ x\ I_{prog}\ x\ T_{prog} \qquad (2)$$

Where T_{prog} is the programming voltage pulse width, V_{prog} is the programming voltage and I is the programming current.

Supplementary Discussion

Supplementary Discussion 1: Effect of metal electrode on ReRAM switching Characteristics

The DC switching cycle of Ag-Ag, Ag-CNT and CNT-CNT electrodes are shown in Figure S8. We observe similar abrupt switching, in all the three cases, irrespective of the metal contacts used. This observation further confirms that the switching mechanism is inherent to the printed WSe_2 material, likely due to Se vacancies and thus ruling out the possibility of Ag ion diffusion. It should be noted that the higher set voltage observed for CNT-CNT electrode based ReRAM is attributed to the thicker switching element formed with 80 passes, as opposed to thinner WSe_2 with 40 passes.

Supplementary Discussion 2: Projection of subthreshold swing with gate dielectric thickness

As the device in the manuscript is not a representative of the current state of the art devices, owing to the large operating gate voltage and large sub threshold swing (*SS*), we have projected sub threshold swing variation with the gate dielectric thickness (Supplementary Figure 12) using the relation, $SS = \frac{kT}{q} ln\, 10 \left(1 + \frac{qDit}{C_{0x}}\right)$. The large gate voltage requirement is due the 90 nm thick SiO_2 gate dielectric. The relatively thicker oxide is chosen to limit the gate current and not to be a detractor in our analysis of the intrinsic channel properties such as conductivity, charge, contact barrier resistance etc of WSe_2 FET after plasma oxidation. The plot shows the contribution of D_{it} (interface trap density) Vs. short-channel electrostatic (SCE) (E.g. source-to-drain tunneling current, source-drain charge sharing for L_g -10 nm) impact on SS. Extensive process optimization with respect to gate stack could potentially improve D_{it}. This is an active area of investigation, which require fundamental material-process co-innovation. To compare the projected device performance with smaller gate length, simulated subthreshold swing for 10 nm gate length device from literature is also added[2,3].

Supplementary References

1. Liu, B. *et al.* High-performance WSe_2 field-effect transistors via controlled formation of in-plane heterojunctions. *ACS Nano* 10, 5153–5160 (2016).

2. Cao, W., Kang, J., Sarkar, D., Liu, W. & Banerjee, K. 2D semiconductor FETs—Projections and design for sub-10 nm VLSI. *IEEE Trans. Electron Devices* 62, 3459–3469 (2015).

3. Resta, G. V *et al.* Scaling trends and performance evaluation of 2-dimensional polarity-controllable FETs. *Sci. Rep.* 7, 45556 (2017).

4. Yoshimine, R., Moto, K., Suemasu, T. & Toko, K. Advanced solid-phase crystallization for high-hole mobility (450 $cm^2 V^{-1}s^{-1}$) Ge thin film on insulator. *Appl. Phys. Express* 11, 31302 (2018).

5. Dürkop, T., Getty, S. A., Cobas, E. & Fuhrer, M. S. Extraordinary mobility in semiconducting carbon nanotubes. *Nano Lett.* 4, 35–39 (2004).

6. Iannaccone, G., Bonaccorso, F., Colombo, L. & Fiori, G. Quantum engineering of transistors based on 2D materials heterostructures. *Nat. Nanotechnol.* 13, 183 (2018).

7. Shih, C. W. & Chin, A. Remarkably High Mobility Thin-Film Transistor on Flexible Substrate by Novel Passivation Material. *Sci. Rep.* 7, 1147 (2017).

8. Hudait, M. K., Clavel, M., Goley, P., Jain, N. & Zhu, Y. Heterogeneous integration of epitaxial Ge on Si using AlAs/GaAs buffer architecture: Suitability for low-power fin field-effect transistors. *Sci. Rep.* 4, 6964 (2014).

9. Shulaker, M. M. *et al.* Monolithic 3D integration: a path from concept to reality. in *Proceedings of the 2015 Design, Automation & Test in Europe Conference & Exhibition* 1197–1202 (EDA Consortium, 2015).

10. Kim, J. H. *et al.* Centimeter-scale Green Integration of Layer-by-Layer 2D TMD vdW Heterostructures on Arbitrary Substrates by Water-Assisted Layer Transfer. *Sci. Rep.* 9, 1641 (2019).

11. Kamiya, T. & Hosono, H. Material characteristics and applications of transparent amorphous oxide semiconductors. *NPG Asia Mater.* 2, 15 (2010).

12. Yamamoto, M., Nakaharai, S., Ueno, K. & Tsukagoshi, K. Self-limiting oxides on WSe_2 as controlled surface acceptors and low-resistance hole contacts. *Nano Lett.* 16, 2720–2727 (2016).

13. Chuang, H.-J. *et al.* Low-resistance 2D/2D ohmic contacts: A universal approach to high-performance WSe_2, MoS_2, and $MoSe_2$ transistors. *Nano Lett.* 16, 1896–1902 (2016).

14. Chuang, H.-J. *et al.* High Mobility WSe_2 p - and n - Type Field-Effect Transistors Contacted by Highly Doped Graphene for Low-Resistance Contacts. *Nano Lett.* 14, 3594–3601 (2014).

15. Son, D. *et al.* Colloidal Synthesis of Uniform-Sized Molybdenum Disulfide Nanosheets for Wafer-Scale Flexible Nonvolatile Memory. *Adv. Mater.* 28, 9326–9332 (2016).

16. Bessonov, A. A. *et al.* Layered memristive and memcapacitive switches for printable electronics. *Nat. Mater.* 14, 199 (2015).

17. Hao, C. *et al.* Liquid-Exfoliated Black Phosphorous Nanosheet Thin Films for Flexible Resistive Random Access Memory Applications. *Adv. Funct. Mater.* 26, 2016–2024 (2016).

18. Huang, Y.-J. & Lee, S.-C. Graphene/h-BN Heterostructures for Vertical Architecture of RRAM Design. *Sci. Rep.* 7, 9679 (2017).

19. Puglisi, F. M. *et al.* 2D h-BN based RRAM devices. in *2016 IEEE International Electron Devices Meeting (IEDM)* 34–38 (IEEE, 2016).

20. Xu, R. *et al.* Vertical MoS_2 Double-Layer Memristor with Electrochemical Metallization as an Atomic-Scale Synapse with Switching Thresholds Approaching 100 mV. *Nano Lett.* 19, 2411–2417 (2019).

21. Zhang, F. *et al.* An Ultra-fast Multi-level $MoTe_2$-based RRAM. in *2018 IEEE International Electron Devices Meeting (IEDM)* 22–27 (IEEE, 2018).

22. Lee, S., Sohn, J., Jiang, Z., Chen, H.-Y. & Wong, H.-S. P. Metal oxide-resistive memory using graphene-edge electrodes. *Nat. Commun.* 6, 8407 (2015).

23. Yan, X. *et al.* Highly improved performance in $Zr_{0.5}Hf_{0.5}O_2$ films inserted with graphene oxide quantum dots layer for resistive switching non-volatile memory. *J. Mater. Chem. C* 5, 11046–11052 (2017).

24. Cheng, L. *et al.* Reprogrammable logic in memristive crossbar for in-memory computing. *J. Phys. D. Appl. Phys.* 50, 505102 (2017).

25. Govoreanu, B. *et al.* 10× $10nm^2$ Hf/HfO_x crossbar resistive RAM with excellent performance, reliability and low-energy operation. in *Electron Devices Meeting (IEDM), 2011 IEEE International* 31–36 (IEEE, 2011).

26. Shih, C.-C. *et al.* Ultra-Low Switching Voltage Induced by Inserting SiO_2 Layer in Indium–Tin–Oxide-Based Resistance Random Access Memory. *IEEE Electron Device Lett.* 37, 1276–1279 (2016).

27. Song, Y. L. *et al.* Low Reset Current in Stacked AlO_x/WO_x Resistive Switching Memory. *IEEE electron device Lett.* 32, 1439–1441 (2011).

28. Breuer, T. *et al.* Realization of minimum and maximum gate function in Ta_2O_5-based memristive devices. *Sci. Rep.* 6, 23967 (2016).

29. Huang, P. *et al.* Reconfigurable nonvolatile logic operations in resistance switching crossbar array for large-scale circuits. *Adv. Mater.* 28, 9758–9764 (2016).
